# Insights into the Regulation of Rice Seed Storability by Seed Tissue-Specific Transcriptomic and Metabolic Profiling

**DOI:** 10.3390/plants11121570

**Published:** 2022-06-14

**Authors:** Fangzhou Liu, Nannan Li, Yuye Yu, Wei Chen, Sibin Yu, Hanzi He

**Affiliations:** 1 College of Plant Science and Technology, Huazhong Agricultural University, Wuhan 430070, China; liufz027@163.com (F.L.); lnn151212@126.com (N.L.); tripley2019@163.com (Y.Y.); chenwei0609@mail.hzau.edu.cn (W.C.); ysb@mail.hzau.edu.cn (S.Y.); 2 Weizhai Town Agricultural Comprehensive Service Station, Fuyang 236418, China; 3 Glbizzia Bioinformatics Institute, Beijing 102629, China; 4 National Key Laboratory of Crop Genetic Improvement, Huazhong Agricultural University, Wuhan 430070, China

**Keywords:** seed storability, transcriptome, metabolome, rice

## Abstract

Non-dormant seeds are continuously aging and deteriorating during storage, leading to declining seed vigor, which is a challenge for the rice seed industry. Improving the storability of seeds is of great significance to ensure the quality of rice and national food security. Through a set of chromosome segment substitution lines population constructed using *japonica* rice NIP as donor parent and *indica* rice ZS97 as recurrent parent, we performed seed storability QTL analysis and selected four non-storable NILs to further investigate the storability regulatory mechanisms underlying it. The seeds were divided into four tissues, which were the embryo, endosperm, aleurone layer, and hull, and tissue-specific transcriptome and metabolome analyses were performed on them. By exploring the common differentially expressed genes and differentially accumulated metabolites, as well as the KEGG pathway of the four non-storable NILs, we revealed that the phenylpropanoid biosynthesis pathway and diterpenoid biosynthesis pathway played a central role in regulating seed storability. Integrated analysis pinpointed 12 candidate genes that may take part in seed storability. The comprehensive analysis disclosed the divergent and synergistic effect of different seed tissues in the regulation of rice storability.

## 1. Introduction

Rice is one of the major food crops in the world, feeding more than half of the world’s population, especially in Asia [1]. As an important agronomic trait of rice, storability measures the germination and viability of rice seeds after storage [2], and improving seed storability is of great significance to ensure the quality of rice and national food security.

Seed storability of rice has extensive variations around the world. Generally, *indica* rice has relatively high storability compared with *japonica* rice [3,4,5,6]. Researchers used natural populations to identify the variation of seed storability and successively detected more than 50 QTLs (Quantitative Trait Loci) of rice seed storability [7]. These QTLs were distributed on every chromosome except chromosome 10, indicating that rice seed storability is a complex quantitative trait controlled by multiple genes. Among them, a QTL located on chromosome 9 was detected in multiple genetic populations [8,9,10], indicating that this locus is a stable locus affecting seed storability. Miura et al. [8] used the recombinant inbred line constructed by NIP and Kasalath to detect *qLG-9* on chromosome 9, explaining the phenotypic variation rate as high as 60%. Li et al. [10] used the BIL (Backcross Inbred Lines) population constructed by Koshihikari and Kasalath and detected a QTL *qSS-9* which is co-located in the same region as *qLG-9*. Later, this research team fine-mapped *qSS-9* to 147 KB [11]. In addition to this locus, the phenotypic variation explained by other QTLs for seed storability is mostly between 6 and 20%, indicating that rice seed storability is a quantitative trait controlled by minor effect genes.

Reactive oxygen species (ROS) play a dual role in seed physiology [12]. On the one hand, as a cellular redox agent, it plays a positive role in regulating seed dormancy, germination, and hormone levels [13]. On the other hand, the accumulation of oxidative damage during seed storage is the main reason for the decline of seed quality. The presence of antioxidants enables seeds to remove excess free radicals during storage [14], including enzyme systems such as superoxide dismutase, catalase, and peroxidase, as well as non-enzymatic systems such as ascorbic acid, vitamin E, and glutathione [15]. Therefore, the seed storability is affected by the balance between reactive oxygen species production and the antioxidant system [16].

1--Cys peroxiredoxin (1--Cys PRX, PER1) is an antioxidant that can eliminate ROS with cysteine residues. Chen et al. [17] found that the storability and abiotic stress tolerance of seeds were enhanced after overexpression of lotus *PER1* in Arabidopsis, indicating that *NnPER1* controls seed longevity by mediating ROS clearance. Studies on this gene in rice showed that the activation of *PER1* in rice seeds was regulated by two bZIP transcription factors (bZIP23 and bZIP42), which could improve seed vigor by scavenging ROS in seeds [18]. In addition, the antioxidant enzyme system in the seed coat also plays a positive role in improving the seed storability [19]. Yuan et al. [20] conducted a QTL analysis on seed storability and seed antioxidant levels. The results showed that they had co-located QTLs. Through the phenotype analysis of transgenic seeds, it was verified that the fatty acid hydroxylase gene (*OsFAH2*) could enhance seed storability by reducing the degree of lipid peroxidation.

In barley, the redox state of glutathione is an important marker of seed deterioration [21]. In Arabidopsis, mutations in vitamin E genes *vte1* and *vte2* lead to vitamin E deficiency and greatly reduce seed longevity [22]. Hwang et al. [23] identified a vitamin E mutant whose tocopherol content was significantly higher than that of the wild type, and its seed vigor and storability were also higher than that of the wild type. Chen et al. [24] constructed RNAi lines with three key genes of vitamin E, the germination rate of all the RNAi lines was significantly lower than that of the wild type, and the seedling growth was poor.

In the early stage of seed development, the hull provides photosynthetic products for the development of internal spikelets through photosynthesis. In the later stage of seed development, the hull can avoid the infection of seeds by germs or pests and reduce the impact of adverse environments. Although there are few studies on the hull affecting seed storability in rice, studies on other plants have shown the potential role of the seed coat and its permeability on seed vigor. In *Arabidopsis thaliana*, increased flavonoid content in the seed coat resulted in increased permeability of the seed coat, which in turn decreased seed dormancy and viability [25,26]. A study of *Taxus yunnanensis* found that chemicals in the seed coat inhibit seed germination, and permeability increases with prolonged storage time [27]. Seed dormancy and storability reflect seed vigor, and these studies suggest a possible relationship between the seed coat and seed storability.

In all, seed storability is regulated by numerous factors. However, as seeds are composed of several tissues, such as embryo, endosperm, seed coat, etc., how those different tissues function together to control seed storability is worth investigating. Therefore, in the present study, we started with QTL mapping of rice seed storability and then selected four non-storable NILs (Near Isogenic Lines) and performed transcriptome and metabolome on different seed tissues. Dry seed is a relatively quiescent stage, and translation is rarely active in dry seeds. However, important metabolites, such as vitamin E and flavonoids, as described above, are stored during maturation and desiccation as a result of the translated important transcripts. Afterward, during dry seed storage, those important transcripts are kept in the dry seeds, as huge transcripts changes can occur only after imbibition [28], and the transcripts in the dry seeds can provide a snapshot of the metabolism that leads to the biochemical and physical features of the dry seeds. Therefore, we performed an integrated analysis of the transcriptome and metabolome. We aim to uncover the function of various seed tissues on rice storability and disclose the important metabolic pathways which regulate seed storability.

## 2. Results

### 2.1. Seed Storability QTL Analysis of NZ CSSL Population

**Figure 1:**
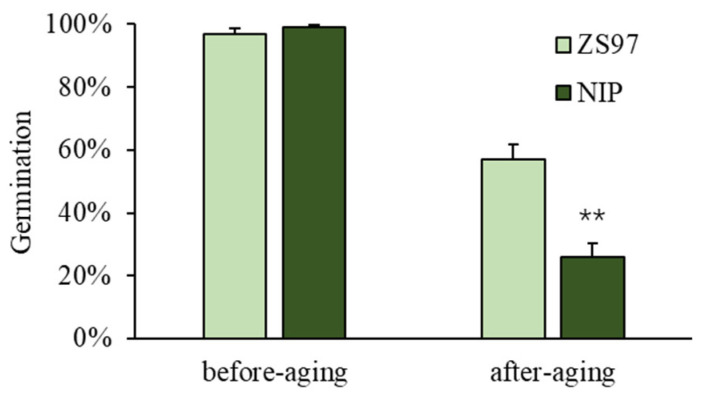
Seed germination phenotype of the two parents NIP and ZS97 before and after artificial aging. ** indicates a significant difference with ZS97 at *p* < 0.01. *p*-values were calculated by Student’s *t*-test.

The typical *japonica* variety Nipponbare (NIP) and the *indica* variety Zhenshan97B (ZS97) significantly differ in seed storability (Figure 1). Before artificial aging, NIP and ZS97 both had high seed vigor, displayed by a high germination percentage (both were higher than 95%). After 8 days of artificial aging, NIP showed a significantly lower seed germination percentage compared with ZS97 (Figure 1). Therefore, the CSSL (Chromosome Segment Substitution Line) population using NIP as donor parent and ZS97 as recurrent parent named the NZ population, including 238 lines utilized for seed storability QTL mapping. Four germination parameters were used to assess seed vigor, the maximum germination percentage after 7 days of germination (Gmax), the germination speed represented by the time it takes for 50% of seeds to germinate (T50), the time it takes for 10% of seeds to germinate (T10), and the area under the germination curve (AUC). AUC is a comprehensive parameter that could reveal both the germination percentage and germination speed collectively.

**Figure 2:**
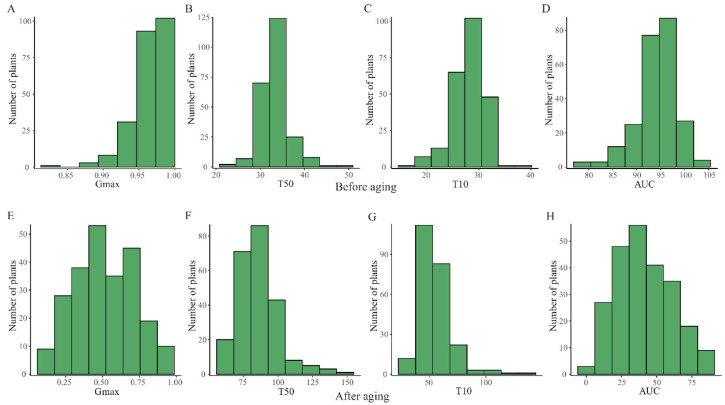
Frequency distribution of the germination parameters before and after artificial aging of the CSSLs population. (**A**) Distribution map of Gmax before artificial aging, (**B**) Distribution map of T50 before artificial aging, (**C**) Distribution map of T10 before artificial aging, (**D**) Distribution map of AUC before artificial aging, (**E**) Distribution map of Gmax after artificial aging, (**F**) Distribution map of T50 after artificial aging, (**G**) Distribution map of T10 after artificial aging, (**H**) Distribution map of AUC after artificial aging.

Gmax of almost all the CSSL population before artificial aging reached higher than 90% (Figure 2A). The germination speed of T50 ranged from 24.3 h to 50.8 h (Figure 2B), T10 ranged from 16.5 h to 38.9 h (Figure 2C), and AUC ranged from 78.2 to 103.3 (Figure 2D), indicating that each line of NZ population had high seed germination uniformity and high seed vigor. After artificial aging of 8 days, Gmax showed a normal distribution ranging from 10% to 91% (Figure 2E). The germination speed of T50 ranged from 58.9 h to 145.5 h (Figure 2F), T10 ranged from 29.2 h to 135.2 h (Figure 2G), and AUC ranged from 3.0 to 88.2 (Figure 2H). Compared with the germination parameters before artificial aging, the germination percentage was decreased, and the germination speed was slowed down with a smaller AUC than the seeds before artificial aging (Figure 2), indicating reduced seed vigor after aging. In addition, these results indicated large genetic variation for seed germination after artificial aging. Thus, the four parameters, Gmax, T50, T10, and AUC, were used to assess seed storability after artificial aging.

**Figure 3:**
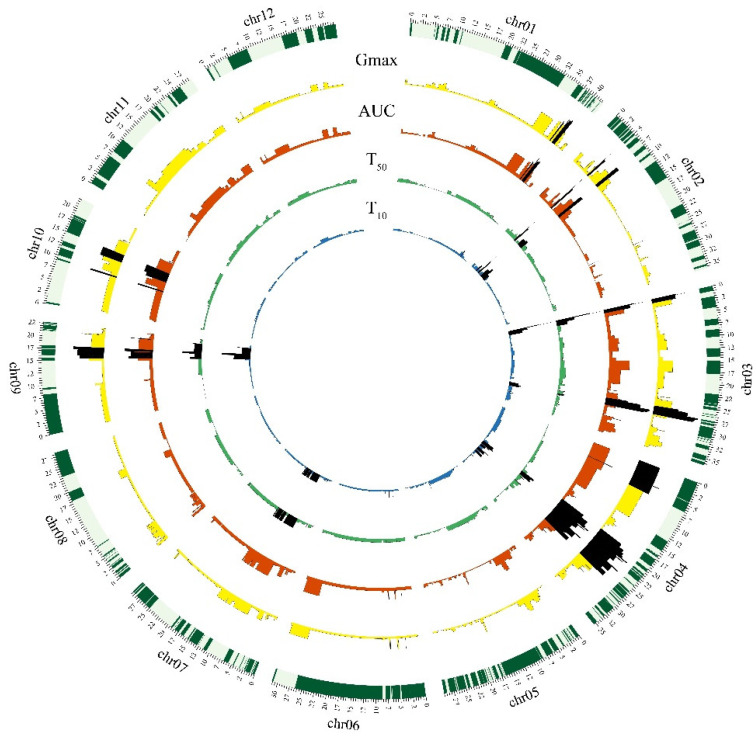
Histogram of Circos, four indicators for evaluating the storage quality of rice seeds. The outermost circle of the Circos diagram is the chromosome marker distribution map. The chromosomes (chr01-chr12) are arranged clockwise. Each sector bar is the size of the chromosome (Mb). The second circle is the Gmax (maximum germination percentage of seven days germination) indicator QTL distribution chart, yellow is *p* > 0.01, the black is *p* ≤ 0.01; the third circle is the AUC (area under the germination curve until 168 h) indicator QTL distribution chart, red is *p* > 0.01, and black is *p* ≤ 0.01; The fourth circle is the T50 (time to reach 50% germination of the total number of germinated seeds) index QTL distribution chart, green is *p* > 0.01, black is *p* ≤ 0.01; the fifth circle is the T10 (time to reach 10% germination of the total number of germinated seeds) index QTL distribution chart, blue is *p* > 0.01, and black is *p* ≤ 0.01. The QTL analysis is calculated by the R language ridge packet RR algorithm.

To explore genetic factors for seed storability, QTL analysis was performed on Gmax, AUC, T50, and T10 after artificial aging. A total of 16 QTLs were detected for seed storability using the four parameters in the CSSL population, with each QTL exhibiting the smallest *p*-value and phenotypic variation explained rate (Figure 3, Table 1). Among the four parameters, T10 explained the highest phenotypic variance (64.9%), with the largest number of QTLs detected (12 QTLs). Gmax, AUC, and T50 explained 52.8%, 52.1%, and 44.3% of the phenotypic variance with 11, 11, and 7 QTLs detected. The 16 QTLs were distributed on the chromosome 1, 2, 3, 4, 5, 6, 7, 9, and 10, in which chromosome 3 had the lowest *p*-value (4.77 × 10^−14^) for T50, and the phenotypic variance was 11.9%.

**Figure 4:**
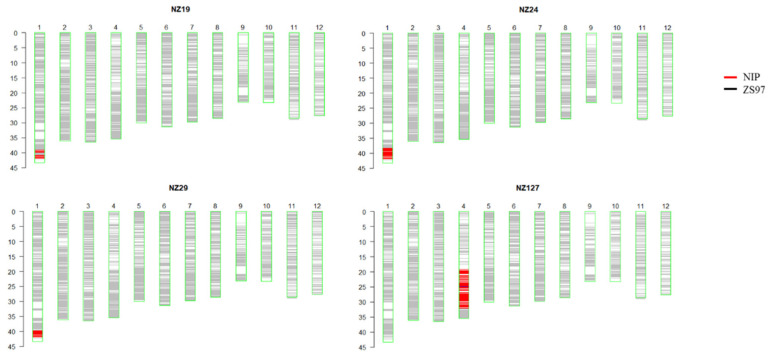
Array chip information of the four selected non-storable NILs. NIP introgressed segment is shown in red, and the gray background is the background material ZS97.

In general, a QTL study should be based on at least two environments (fields or years), but in the present study, it was essentially used to identify contrasting lines and loci for further studies (profiling); therefore, only one year of data was used for QTL study. Among all the identified seed storability QTLs, *qSST1.2* were detected in Gmax and AUC, and three NILs (NZ19, NZ24, and NZ29) contained this QTL region and had a very small introgression region. *qSST4.2* had the highest PVE (17.2%). The near-isogenic line NZ127 contained the *qSST4.2* region (Figure 4). Therefore, those four lines (NZ19, NZ24, NZ29, and NZ127) that contain these two QTLs were selected from the CSSL population for further study. The detailed introgression segment positions are listed in Table S1. Based on Gmax and AUC, the seed storability of the four selected lines was significantly lower than the background line ZS97 (Table 2).

**Figure 5:**
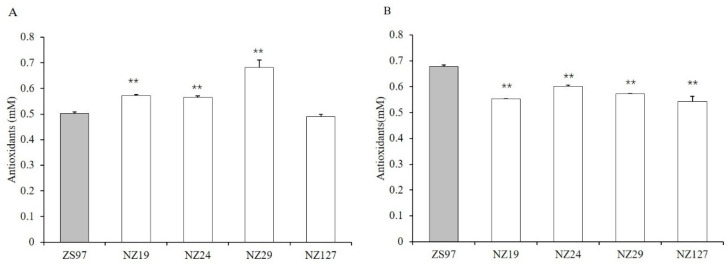
Total antioxidant content in rice seeds. (**A**) Total antioxidant content of unaged rice seeds; (**B**) Total antioxidant content of rice seeds after artificial aging. ** indicates significant difference at *p* < 0.01. *p* values were calculated by Student’s *t*-test.

The reduction of seed storability is often associated with the oxidation of cellular macromolecules such as nucleic acids, proteins, and lipids [2]. Therefore, antioxidant levels were measured to assess the seed storability (Figure 5). Before artificial aging, NZ19, NZ24, and NZ29 had significantly higher levels of total antioxidant content, while NZ127 had no difference compared with ZS97. After artificial aging, all four lines had significantly lower levels of antioxidants (Figure 4, Table 2). The imbalance between the production and scavenging of reactive oxygen species is the main cause of seed aging. As the storability of ZS97 was significantly higher than the other four NILs, we speculated that ZS97 produced less ROS and the ROS scavenging system (which was measured by the antioxidant levels in Figure 4) would be at a low level compared with the NILs. Afterward, if the seed was stored for a long time or in unsuitable conditions (i.e., high temperature, elevated relative humidity), the accumulation of cellular oxidative damage progressively induced a loss of seed vigor. To promote their longevity, seeds require efficient antioxidant systems [2]. Consequently, after aging, ZS97 had higher antioxidants than before aging, and the more storable ZS97 contained higher levels of antioxidants than the four non-storable NILs.

### 2.2. Extensive Differential Gene Expression Occurs in Distinct Rice Seed Tissues in Selected NILs

**Figure 6:**
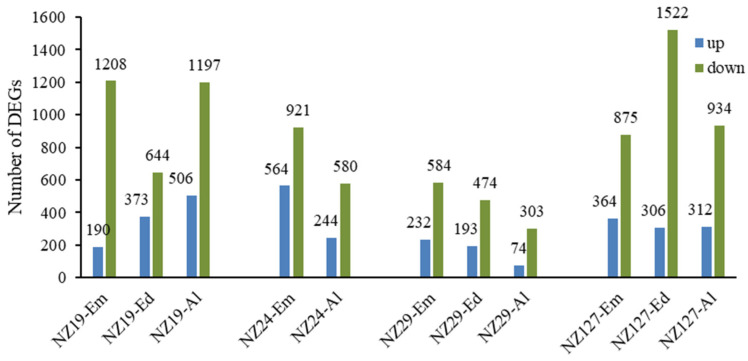
The number of DEGs (differentially expressed genes) in four NILs compared with ZS97. Em, Ed and Al represent embryo, endosperm, and aleurone layer.

To explore the genetic mechanisms behind seed storability in detail, we performed transcriptome analysis in three rice grain tissues (embryo, endosperm, and aleurone layer) of non-aged grain with the selected four NILs (NZ19, NZ24, NZ29, and NZ127) and the background line ZS97. Differentially expressed genes (DEGs) of each tissue between NILs and ZS97 are shown in Figure 6. For all three NILs, in three types of tissues, the number of down-regulated genes was much higher than the number of up-regulated genes. Not surprisingly, the highest number of DEGs was found in NZ127, which had larger NIP introgressions than the others (Figure 6).

The DEGs of each material were enriched with KEGG (Kyoto Encyclopedia of Genes and Genomes) to find their own storage regulatory pathways. DEGs in NZ19 embryos are mainly enriched in diterpenoid biosynthesis, zeatin biosynthesis, and starch and sucrose metabolism; DEGs in NZ24 embryos were mainly enriched in diterpenoid biosynthesis, phenylpropanoid biosynthesis pathway, phenylalanine metabolism pathway, and zeatin biosynthesis pathway; DEGs in NZ29 embryos are mainly enriched in phenylpropanoid biosynthesis pathway, phenylalanine metabolism pathway; DEGs in NZ29 endosperm are mainly enriched in diterpenoid biosynthesis pathway and phenylpropanoid biosynthesis pathway; the DEGs in the aleurone layer of NZ29 are mainly enriched in diterpenoid biosynthesis pathways. Although NZ127 contained a large introgressed fragment of NIP and had the highest number of DEGs, there was no enriched KEGG pathway for all three tissues (Figure S1).

Therefore, the KEGG pathways of DEGs of each substitution line were unique, but they were mainly enriched in the phenylpropanoid biosynthesis pathway and diterpenoid biosynthesis pathway, indicating those two pathways were greatly involved in regulating rice seed storability.

### 2.3. Key DEGs about Cell Wall Building, Energy Supplement, and Hormone Synthesis Were Detected to Be Tightly Associated with Seed Storability

**Figure 7:**
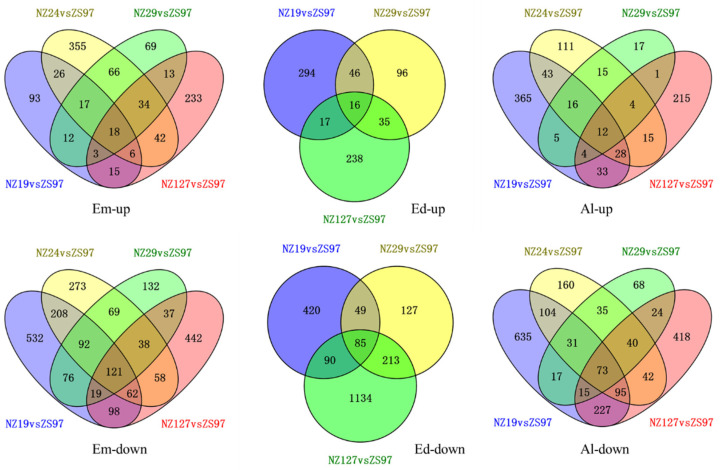
Venn diagram of differential expressed genes between 4 selected NILs (NZ19, NZ24, NZ29, NZ127) and ZS97. Em, Ed, Al, and Hu represent embryo, endosperm, aleurone layer, and hull.

To identify genes showing a similar expression pattern in the four non-storable NILs, the common DEGs in these genotypes were obtained and displayed in the form of Venn diagrams (Figure 7). As the number of down-regulated genes was higher than up-regulated genes, the commonly down-regulated genes in the four non-storable NILs were also more than the commonly up-regulated genes in all the genotypes. The common DEGs in the four non-storable NILs are listed in Table S2.

Next, in order to identify gene categories that were responsible for rice seed storability, we investigated the common DEGs in the four non-storable NILs by searching for annotation information, functions of orthologous genes in other species, and related literature. In commonly up-regulated genes of four genotypes in all three tissues, there were genes annotated with histones and their components, while the commonly down-regulated genes could be divided into seven categories based on their functions, including hormone-related, cell wall remodeling, energy supplement, efflux transportation, seed germination, and stress-related genes (Table 3).

Through comparison among three tissues (embryo, endosperm, and aleurone layer), we found that some differentially expressed genes were tissue-specific while some genes were identified in multiple tissues. For example, the types of hormones and the related genes were different in the three types of tissues (Table 3). In the embryo, the ABA-related gene *HVA22* was commonly down-regulated in the four NILs, while the other three ABA-related genes (*OsASR5*, *OsASR6*, and *GEM*) were commonly down-regulated in the endosperm, and the BR-related gene *OsDWARF* was commonly down-regulated in the aleurone layer. As for cell wall-related genes, there was one gene in the embryo (expansin precursor), one gene in the endosperm (glycosylhydrolase family), and two genes in the aleurone layer (*CESA6* and *Os4CL5*) commonly down-regulated in the aleurone layer of the NILs. The distribution of these differentially expressed genes with various functions in unique tissues shows the tissue specificity of rice seeds.

In addition to the tissue-specific genes, several common differentially expressed genes were identified in multiple tissues. Two α-amylase-related genes and two types of hormone-related genes (*OsNCED1* and *OsGH3.8*) were commonly down-regulated in two tissues (Table 3), and one stress-related gene (*OsHsfA2a*) was down-regulated in all three tissues, indicating the importance of this gene in seed storability regulation.

### 2.4. Metabolic Analysis and Key Metabolite Identification

**Figure 8:**
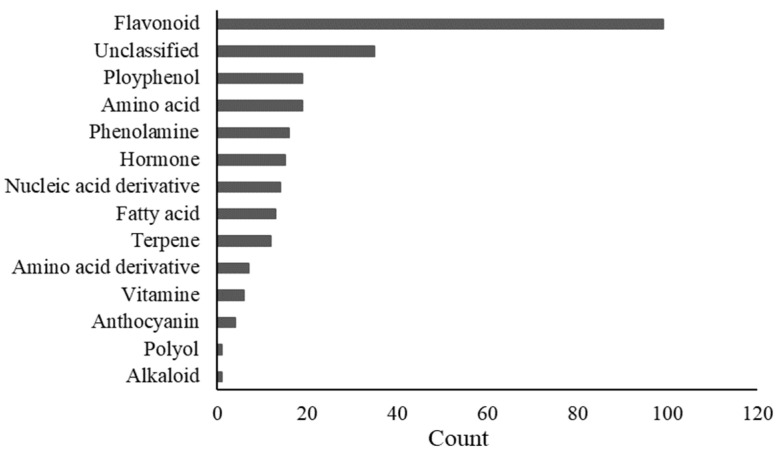
Classification of identified 261 metabolites.

To assess the effects of gene expression in rice seeds on overall metabolism, metabolite profiling of the embryo, endosperm, aleurone layer, and hull of four non-storable NILs and the background line ZS97 was used to perform LC-ESI-MS/MS analysis. A total of 261 metabolites were identified, including 99 flavonoids (Figure 8).

**Figure 9:**
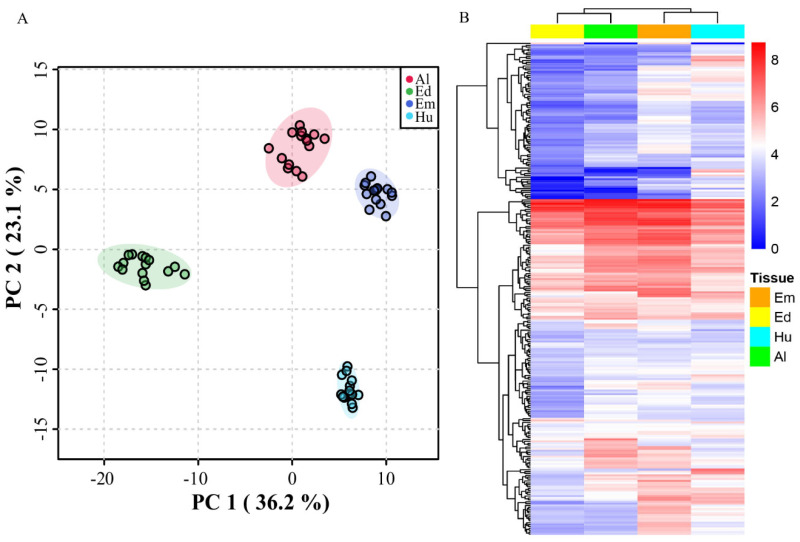
General analysis of metabolites in four non-storable NILs and ZS97. PCA (**A**) and heatmap (**B**) of all the metabolites. Em, Ed, Al, and Hu represent embryo, endosperm, aleurone layer, and hull, respectively.

PCA analysis showed that the metabolites in the five genotypes were separated according to the different seed tissues (Figure 9A). The background ZS97 cannot be distinguished clearly from the other non-storable NILs, indicating that, on the metabolites level, there were more differences between seed tissues than seed storability. According to the heatmap, most of the metabolites in the endosperm had clearly lower amounts than in other tissues (Figure 9B).

**Figure 10:**
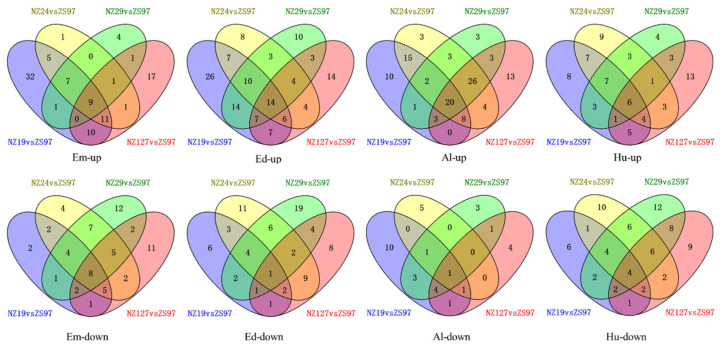
Venn diagram of different metabolites between four selected NILs (NZ19, NZ24, NZ29, NZ127) and ZS97. Em, Ed, Al, and Hu represent embryo, endosperm, aleurone layer, and hull.

Differentially accumulated metabolites were calculated using |log2FC| > 1 and *p*-value < 0.05 for the NILs compared with ZS97. In contrast with the transcriptome analysis, the Venn diagram analysis revealed that the number of significantly increased common metabolites in four non-storable NILs was higher than the significantly decreased common metabolites (Figure 10), and the common metabolites between materials were mainly focused in flavonoids and phenolamine.

There were 9 metabolites commonly increased in seed embryos of 4 non-storable NILs, including 4 flavonoids; 14 metabolites commonly increased in endosperm with 7 flavonoids; 21 metabolites commonly increased in the aleurone layer, including 11 flavonoids, and only 6 increased metabolites in the hull, including 5 flavonoids and 1 polyphenol. However, those increased flavonoids were all tissue-specific; no common flavonoid was found in distinct tissues.

The commonly decreased metabolites in seed embryos contained 3 phenolamines: Np-coumaroylagmatine, N-caffeoylputrescine; a flavonoid (C-hexosyl-luteolin O-p-coumaroylhexoside) was decreased in four NILs in endosperm while another flavonoid, tricin 4′-O-(β-guaiacylglyceryl) ether was decreased in four NILs in the aleurone layer. There was one vitamin (thiamin) commonly decreased in the hull.

Some commonly increased/decreased metabolites in the four non-storable NILs were found in multiple tissues. For example, 4,6-dihydroxyquinoline O-hexoside was decreased in the embryos and hulls of non-storable materials compared to ZS97, and phellodenol H was increased in the seed embryos and aleurone layers of non-storable materials. At the same time, some other metabolites in different tissues showed the opposite regulation trend. For example, di-C, C-hexosyl-methylluteolin was increased in embryos but decreased in the endosperm of non-storable materials; roseoside was decreased in embryos but increased in endosperm; serotonin was decreased in embryos but increased in the aleurone layer, and trans-zeatin N-glucoside was decreased in the hull but increased in the aleurone layer.

### 2.5. DEGs and Metabolites Were Co-Location on Phenylalanine Pathway and Diterpenoid Synthesis Pathway by KEGG

In the KEGG pathways of DEGs analysis, the overall trend of the four non-storable NILs revealed that phenylpropanoid biosynthesis and diterpenoid biosynthesis pathways were significantly enriched (Figure S1). Therefore, the differential metabolites were mapped on those two pathways to explore the genes and metabolites regulation manner.

**Figure 11:**
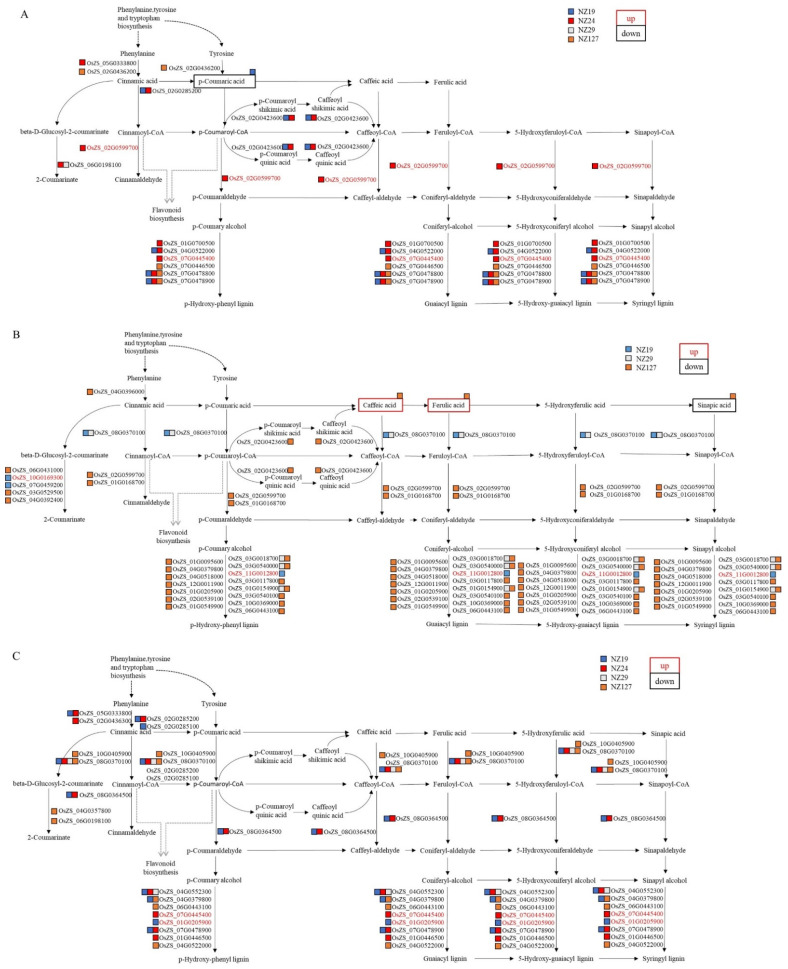
Differential expressed genes and differential accumulated metabolites mapped on phenylpropanoid biosynthesis pathway of embryo (**A**), endosperm (**B**), and aleurone layer (**C**).

In seed embryos, the metabolite p-coumaric acid was significantly decreased in NZ19 relative to ZS97 (Figure 11A) in the phenylpropanoid synthesis pathway. At the same time, 13 differentially expressed genes were detected in this pathway; 11 genes were down-regulated while only 2 genes were up-regulated, OsZS_02G0599700 (LOC_Os02g56680, dehydrogenase) and OsZS_07G0445400 (LOC_Os07g44440, peroxiredoxin) (Figure 11A).

In endosperm, the metabolites caffeic acid and ferulic acid were significantly increased, while the metabolite sinapic acid was significantly decreased in NZ127 in the phenylpropanoid synthesis pathway. Twenty-six differentially expressed genes were located in the same pathway. Only OsZS_11G0012800 (LOC_Os11g02130, peroxidase precursor) was up-regulated in NZ19 relative to ZS97, while all the other genes were down-regulated genes, and most of them were differentially expressed genes detected in NZ127 (Figure 11B).

Eighteen differentially expressed genes in the phenylpropanoid synthesis pathway were detected in the aleurone layer, and most of them were down-regulated genes. Among them, OsZS_08G0370100 (LOC_Os08g34790, *Os4CL5*), which regulates the acylation of coumaric acid, was identified in NZ19, NZ24, NZ29, and NZ127 (Figure 11C), but no differential metabolites in the phenylpropanoid synthesis pathway were detected in the aleurone layer.

The diagram shown is based on KEGG pathway maps (https://www.genome.jp/kegg/ accessed on 20 April 2022). Differential accumulated metabolites are marked with boxes; the red boxes represent increased metabolites levels, the black boxes represent decreased metabolites levels; the differential expressed genes are shown in red font for up-regulation and black font for down-regulation. Different colored squares next to differential expressed genes or differential accumulated metabolites indicate the material where the difference is (the color schemes are shown in the upper right corner of each figure).

**Figure 12:**
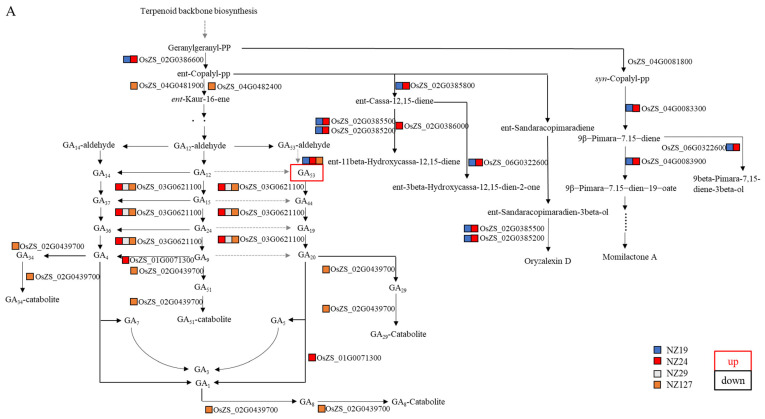

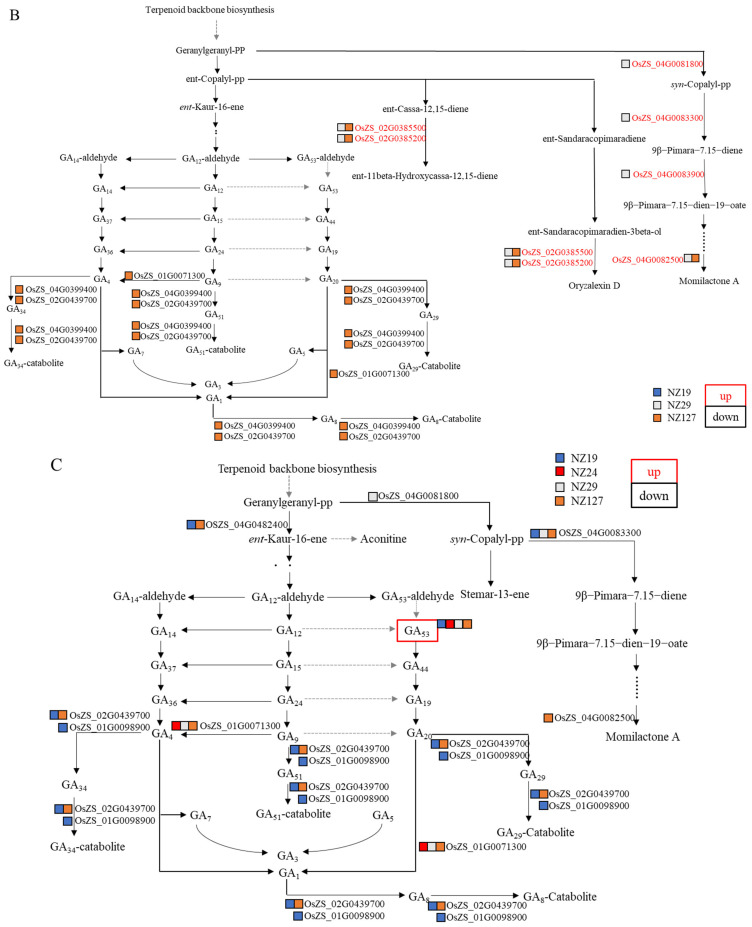
Differential expressed genes and differential accumulated metabolites mapped on diterpenoid biosynthesis pathway of embryo (**A**), endosperm (**B**), and aleurone layer (**C**).

For the diterpenoid synthesis pathway, the differential accumulated metabolite GA53 in the embryos of NZ19, NZ24, and NZ127 was accompanied by 14 differentially expressed genes in this pathway, and these differential genes were all down-regulated genes (Figure 12A), while in the aleurone layer, GA53 was significantly increased in all the 4 non-storable NILs, and 8 down-regulated genes were co-located in the same pathway (Figure 12B). On the other hand, in the endosperm, the GA (gibberellic acid) levels were not changed in metabolites analysis in the diterpenoid synthesis pathway, although there were 9 differential genes in the pathway and the genes responsible for the production of various forms of GA were all significantly down-regulated (only in NZ127) (Figure 12C). These results pointed out the significance of GA in regulating seed storability in the embryo and aleurone layer.

The diagram shown is based on KEGG pathway maps (https://www.genome.jp/kegg/ accessed on 20 April 2022). Differential accumulated metabolites are marked with boxes; the red boxes represent increased metabolites level, the black boxes represent decreased metabolites level; the differential expressed genes are shown in red font for up-regulation and black font for down-regulation. Different colored squares next to differential expressed genes or differential accumulated metabolites indicate the material where the difference is.

### 2.6. Candidate Gene Prediction

Since each NIL line has only one/two small introgressed segments from NIP into ZS97, the generation of differentially expressed genes or metabolites is theoretically caused by the introgressed NIP fragment. The NILs were genotyped using an Infinium RICE8K array (Illumina) chip [29]. In order to explore the key genes that affect the storability in the introgressed fragments, we combined transcriptome and metabolome information together with the array chip information. Two methods were used to identify candidate genes, which were common differentially expressed genes (differentially expressed genes in the selected four NILs) and differentially expressed genes involved in key KEGG pathways that fell in the introgressed segment (Table 4).

There were six common differentially expressed genes that fell in the introduced segment, all of which were down-regulated. Except for one gene, LOC_Os01g71860, located in NZ19, NZ24, and NZ29, the remaining five genes all fell into the introduction section of NZ127. Among them, LOC_Os01g71860 and LOC_Os04g33640 were both annotated as glycosyl hydrolases family 17.

For the genes in the key KEGG pathway (phenylpropanoid biosynthesis pathway and diterpenoid biosynthesis pathway), there were six genes that fell in the introgressed segments, all of which were down-regulated genes and all fell in the introgressed segment of NZ127. Two out of those six genes were beta-glucosidase-related genes (LOC_Os04g39864 and LOC_Os04g43410), and one gene was involved in the GA pathway (gibberellin 2-oxidase gene), which converts the GA active form into an inactive form. Based on the above information, we believe that these 12 genes were most likely to be the key genes that cause the ono-storable phenotype of the 4 selected NILs compared to ZS97.

We selected 4 genes from these 12 genes (LOC_Os04g52210, LOC_Os01g71860, LOC_Os04g44580, and LOC_Os04g52504) and performed qPCR verification in NZ127. We found that the expression levels of these candidate genes in non-storable NILs were indeed reduced by more than two times compared with ZS97 (Figure S2).

**Figure 13:**
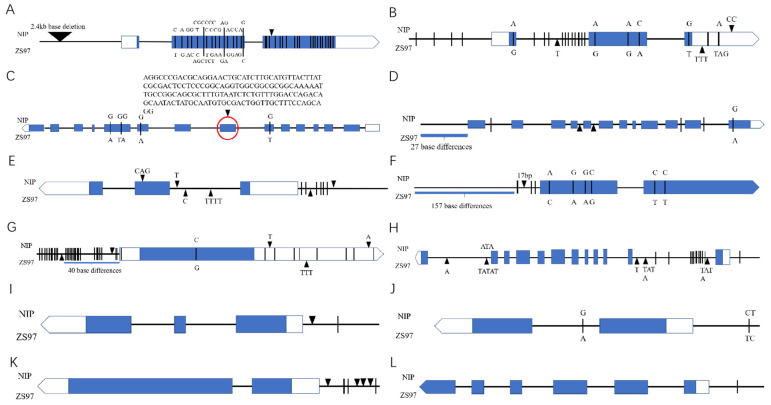
Schematic diagram and sequence variation of 12 candidate genes. (**A**) LOC_Os01g71860; (**B**) LOC_Os04g33640; (**C**) LOC_Os04g39864; (**D**) LOC_Os04g52210; (**E**) LOC_Os04g52504; (**F**) LOC_Os04g53630; (**G**) LOC_Os04g48290; (**H**) LOC_Os04g43410; (**I**) LOC_Os04g44500; (**J**) LOC_Os04g44150; (**K**) LOC_Os04g43800; (**L**) LOC_Os04g44580.

In order to further verify the candidate genes, we analyzed the sequence differences of the 12 candidate genes between the 2 parents, NIP and ZS97 (Figure 13), and found that 7 genes (LOC_Os01g71860, LOC_Os04g33640, LOC_Os04g39864, LOC_Os04g48290, LOC_Os04g52210, LOC_Os04g52504, and LOC_Os04g53630) had variations in coding regions. The other five genes only had sequence variation on the promoter and/or intron. Protein sequence alignment demonstrated that only four genes (LOC_Os01g71860, LOC_Os04g39864, LOC_Os04g52504, and LOC_Os04g53630) had variations in the protein sequence. These variations may be the causal factor for the storability differences between the NILs and ZS97.

The blue box represents exons; the blank arrow represents 3′-UTR. The black triangle represents INDEL (triangle indicates insertion in NIP while inverted triangle indicates insertion in ZS97 sequence). The black line represents SNP.A, B, C, D, E, F, G: Genes with changes in the coding region (with changes at the protein level). H, I, J, K, L: Genes with changes in promoters/introns.

## 3. Discussion

### 3.1. Divergent and Synergistic Effect of Different Seed Tissues in the Regulation of the Storability of Rice

Rice grain is composed of several tissues, including the embryo, endosperm, aleurone layer, and hull. In the present study, by investigating the regulation mode of different grain tissues on genes and metabolites levels of the four non-storable rice materials, we revealed that there were divergent and synergistic effects of different tissues in the regulation of the storability of rice.

In the view of differentially expressed genes, there were three genes related to energy supply commonly down-regulated in the four non-storable NILs, which were all α-amylase-related genes (Table 3). The three genes were all identified in the endosperm; however, two of them, LOC_Os09g28400 and LOC_Os02g52710, were also identified in the embryo and aleurone layer, respectively, while LOC_Os02g52700 was only identified in the endosperm. On the one hand, this result indicated the specificity of endosperm for energy supply; on the other hand, it designated the synergistic effect of endosperm, aleurone layer, and embryo on the gene regulation of α-amylase. In addition to energy supply, the down-regulation of cell wall-related genes in three seed tissue may indicate that the three tissues need to be coordinated for cell wall degradation (Table 3). Interestingly, the down-regulation of a heat shock protein gene, OsHsfA2a, was observed in all three tissues (Table 3). The heat shock protein gene OsHsfA2a, which regulates protein folding and stability and responds to heat stress and oxidative stress, may be related to storability [30].

At the level of metabolites, different tissues presented diverse patterns for the increased and decreased metabolites. The number of increased metabolites in the endosperm was nearly twice the number of decreased metabolites, and this number was five times higher in the aleurone layer, while the number of increased and decreased metabolites in seed embryos and the hull was basically the same.

As for metabolic pathways, there were also differences in the types of genes and metabolites in specific metabolic pathways between different tissues (Figure 12, Figure 13 and Figure S1), indicating different regulatory modes. For example, the gene OsZS_02G0599700 for cinnamaldehyde is up-regulated in seed embryos but down-regulated in the endosperm (Figure 11A,B). Another example is that GA53 was increased in embryos and aleurone layers in the diterpenoid synthesis pathway, but there was no difference in the endosperm. Therefore, it is necessary to study seed storability from the perspective of tissue specificity.

### 3.2. The Importance of Phenylpropanoid Synthesis Pathway in Seed Storability Regulation

The KEGG co-localization of differentially expressed genes and the differential accumulated metabolites pointed out the importance of the phenylpropanoid synthesis pathway (Figure 11). The phenylpropanoid synthesis pathway is the leading pathway for the synthesis of flavonoids and lignin. It is related to proanthocyanidins in the seed coat. Permeability of the seed coat is one of the factors influencing seed storability [31]. The seed coat is the bridge between the seed embryo and the external environment and provides protection for the inner seed content. This protection is mainly determined by the polysaccharides, polyphenols, suberin, and cutin in the seed coat [2,32].

Among these metabolites, polyphenols include flavonoids and lignin. The relationship between flavonoids and seed life is mostly concentrated in the TT (TRANSPARENT TESTA) family. Mutants of these genes had a significantly shallower seed coat due to the inhibition of proanthocyanidin synthesis [33] and showed a significantly lower germination rate compared to the control after being stored at room temperature for 4 years [34], indicating the positive regulation of TT family genes on storability. The relationship between lignin and seed storability is mainly related to the regulation of the structure of the cell wall. Plant secondary cell walls are composed of cellulose, hemicellulose, and lignin. The thickened cell wall strengthens the protection of the seed and reduces the permeability, thereby affecting the penetration of external moisture and oxygen to the embryo during seed germination.

In addition, we detected that the expression of the *Os4CL5* gene in the aleurone layer of non-storable samples was down-regulated significantly (Table 3). 4CL5 is 4-Coumarate-CoA Ligase 5, which is a key enzyme in the pathway of phenylpropanoid synthesis. It catalyzes hydroxycinnamic acid to generate various thioesters and controls the pathway of phenylpropanoid metabolism in different directions [35,36,37]. The thioester catalyzed by 4-Coumarate-CoA Ligase is the precursor for the biosynthesis of lignin and flavonoids [38]. Integrating other genes that control cell wall synthesis (Table 3) (such as cellulose synthase gene *CESA6*, cell wall protein gene *LOC_Os01g71850*, and expansion protein gene *LOC_Os02g51040*, etc.), we believe that the lignin metabolism pathway is related to storability. Indeed, a potential relationship between lignin and seed germination has been reported. In *Arabidopsis thaliana*, 2 mutants for a single laccase gene, *AtLAC15*, reduced extractable lignin content by nearly 30% compared to wild type and showed a lower germination rate [39]. Renard et al. [31] detected changes in total polyphenol content derived from suberin and/or lignin in seeds of gain-of-function mutant *cog1-2D* with increased seed longevity and displayed a thinner palisade layer. The down-regulation of the expression of these cell wall-related genes and metabolites may be caused by reducing the mechanical strength and air or water permeability of the cell wall, which leads to a faster decline in the vitality of the NILs during the aging process.

### 3.3. The Importance of Diterpenoid Synthesis Pathway in Seed Storability Regulation

The integrated analysis also revealed the significance of the diterpenoid synthesis pathway, especially the GA pathway (Figure 12). In our study, the increase of GA53 in the diterpenoid synthesis pathway was detected in the non-storable materials, indicating that GA53 may play a negative regulatory role in the regulation of seed storability. There has been little research that demonstrated the importance of GA with storability; our research provides some future directions to investigate the relevance of GA in the regulation of seed storability.

In addition, GA may affect storage stability by affecting the synthesis of α-amylase. The degradation of starch in the endosperm is the main energy supply pathway for the germination of rice seeds. The sucrose hydrolased from starch is transported to the embryo and is quickly consumed by the growing radicle. In this process, α-amylase plays a key role [40,41]. In our study, the three α-amylase genes *RAmy1A*, LOC_Os02g52700, and LOC_Os09g28400 were reduced by more than two times compared with ZS97, which leads to insufficient energy supply during germination. This could be one of the reasons that the non-storable NILs had lower Gmax and slower germination speed (Table 2). However, as the enzyme activity of α-amylase is triggered by the GA signal, the higher GA53 together and lower expression level of α-amylase is difficult to explain. Therefore, this study provides a new potential research direction for seed storability.

Regulation of hormones on seed germination is a research hotspot, especially for the balance of ABA and GA. As is known, GA inhibits dormancy and promotes germination, while ABA plays the opposite role [42]. Several ABA-related genes were down-regulated, such as *OsNCED4,* two ABA abscisic stress-ripening (ASR) genes (*OsASR5* and *OsASR6*), and *HVA22*. These genes displayed various functions for ABA biosynthesis, dehydration and stress response, xylem structure, etc. [43,44,45,46,47,48,49,50,51]. The role of ABA in seed maturation and storability has been previously identified [3,18,52]. In legumes, ABSCISIC ACID INSENSITIVE5 (ABI5) regulated late seed maturation by influencing longevity and RFO accumulation [53]. In rice, it was found that *OsHIPL1* mutants, which regulated seed vigor, had higher levels of endogenous ABA in germinated seeds, with two ABA biosynthesis genes (*OsZEP* and *OsNCED4*) up-regulated and one ABA catabolism gene, *OsABA8ox3* down-regulated [54]. Our study revealed that they might have a potential function in seed storability regulation by affecting hormone homeostasis.

### 3.4. Prediction of Candidate Genes for Rice Storability

In order to identify candidate genes responsible for seed storability, we integrated transcriptome, metabolome, and array chip information. By analyzing common differentially expressed genes for 4 non-storable NILs and the differentially expressed genes enriched in the key KEGG pathway fell into the NIP-introduced segments, 12 candidate genes were screened out (Table 4).

The sequence analysis results showed that the coding regions of 7 of the 12 candidate genes produced non-synonymous mutations in the parents, resulting in amino acid variation (Figure 13). There were two genes annotated as β-glucosidase (BGlu) related genes (LOC_Os04g39864, LOC_Os04g43410). It was reported that rice β-Glucosidase *Os4BGlu14* (LOC_Os04g43360) negatively affected seed longevity during accelerated aging. The overexpressing (OE) lines displayed a significantly lower germination percentage than the wild type and had higher lignin accumulation before and after accelerated aging [55]. However, in our data, we showed that in non-storable NILs, two BGlu-related genes were down-regulated, indicating a positive regulation of seed storability. Probably seeds need multiple functions of β-glucosidases to balance the seed storability.

There were two members of the glycosyl hydrolase family (LOC_Os01g71860, LOC_Os04g33640) (Table 4). These genes are related to the decomposition of lignocellulose and may influence the storability of rice seeds by regulating the cell wall components. These are worthy of performing follow-up in-depth study.

GA 2-oxidase (GA2ox) (LOC_Os04g44150) is a gene that can inactivate the upstream bioactive GA1/GA4 [56], the down-regulation of this gene could explain the elevated GA53 level in the non-storable NILs (Figure 12). However, we still need more direct evidence to prove the involvement of seed storability.

The candidate gene prediction provides us with new research directions to dive into the molecular mechanisms of seed storability.

## 4. Materials and Methods

### 4.1. Plant Materials

The CSSL population named N/Z used in this study was developed previously using a marker-assisted selection backcross approach in which the recurrent parent was *indica* variety ZS97 and the donor parent was the *japonica* variety Nip. Each line contained a substituted donor segment of a particular chromosomal region within the common background of ZS97, and all the segments together covered most of the donor genome in the CSSL population. The plant materials were cultivated in the experimental field of Huazhong Agricultural University at Wuhan (30.4_N, 114.2_E). They reached maturity around the same date and were immediately harvested and then dried to break dormancy at 43 °C in the oven (101-34B, TAISETE, Tianjin, China) for 5 days to ensure the maximum germination rate of all materials was consistent and reached more than 80%. Then, the materials were equilibrated in a storage chamber with low relative humidity (30% RH) for 4 weeks to obtain a constant moisture content of approximately 12%.

### 4.2. Artificial Aging

To measure seed storability rapidly, artificial aging experiments were conducted using a modified method [3,20,57]. The seeds were stored in a thermostatic moisture regulator (LRHS-400F-II, Longyue, China) at high temperature (43 °C) and RH (95%) for 8 days (pilot experiment of aging for 7 d, 8 d, 9 d, and 11 d on two parents (ZS97 and NIP) were carried out, according to the germination results after aging the artificial aging time, 8 d was determined) and then used for germination experiment.

### 4.3. Germination Experiment

A total of 50 seeds were spread on wetted white filter papers in Petri dishes for seed germination. The experiment was carried out in a 25 °C incubator under 16 h light conditions. The germinated seeds were counted every day. The germination parameters were calculated using the software package “GERMINATOR” [58]. This package allows the extraction of 4 biologically relevant parameters from the germination-time curve to interpret germination performance, including the maximum percentage of germination (Gmax), time to reach 50% germination of the total number of germinated seeds (t50), time to reach 10% germination of the total number of germinated seeds (t10), and integration of the area under the curve (AUC), which provides a value that combines information on Gmax, t50, and uniformity.

### 4.4. QTL Mapping

Based on the SNP genotypes, a bin was defined by a unique overlapping substitution segment from the CSSLs [59] and used as a marker for QTL analysis. A linear ridge regression in the R package “ridge” (http://www.r-project.org/ accessed on 20 April 2022) was applied for QTL analysis in the CSSLs population [60]. A *t*-test for the ridge regression coefficients was conducted for each bin, which was taken as an independent variable in the linear ridge regression model. A significance level of *p* < 0.01 was set as the threshold to declare the presence of a putative QTL in a given bin. If several adjacent bins showed significant *p*-values, then the QTL was tentatively located in the most significant bin with the lowest *p*-value. The variance explained by each QTL (bin) was calculated using lmg by R package “relaimpo”. QTL nomenclature followed the principles suggested by McCouch and CGSNL (2008).

### 4.5. Seed Tissue Separation

Grains were dehulled with a rice huller (JLG-II, Institute of Grain Storage, Chengdu, China), and the embryos were dissected with a sharp knife. The aleurone layer and endosperm were separated using a laboratory bench rice polishing machine (JMNJ-3/CPC 96-3, Hangzhou Qianjiang Instrument Corporation, Hangzhou, China) for 15 s, and then each part of the seeds was milled respectively by a grinder (MM400, Retsch, Haan, Germany) with liquid nitrogen.

### 4.6. Total Antioxidant Content Determination

A total antioxidant capacity (ABTS) detection kit (Nanjing Jiancheng Institute of Bioengineering, Nanjing, China, A015-2) was used to determine the total antioxidants in brown rice. Accurately weigh the rice brown rice, add 9 times the volume of normal saline in the ratio of weight (g): volume (mL) = 1:9, and mechanically homogenize it in an ice-water bath to fully break the cells and release the antioxidants in them, 4 °C, 12,000 rpm, centrifugation for 5 min, take the supernatant for determination. After adding the sample, the reaction was carried out at room temperature for 6 min, the wavelength was 405 nm, and the OD value of each well was measured by a multi-function microplate reader (Spark 10M).

### 4.7. RNA Isolation

RNA in the embryo and aleurone layer was constructed using RNA prep Pure Plant Plus Kit (DP441, TIANGEN, Beijing, China). RNA was isolated from the endosperm using TaKaRa MiniBEST Plant RNA Extraction Kit (NO.9769, TAKARA, Kusatsu, Japan).

### 4.8. Transcriptome Profiling

Transcriptome analysis was performed in the mature, dry rice grain before aging in the three grain tissues (embryo, endosperm, and aleurone layer) with the selected four NILs (NZ19, NZ24, NZ29, and NZ127) and the background line ZS97. The libraries were produced and sequenced by Illumina HiSeq 4000/NovaSeq sequencer. Raw sequences were filtered to remove the adaptor sequences, low-quality (reads containing sequencing Ns > 10%), and short reads (Q < 10 nt), and the resulting sets of high-quality clean reads were used for transcriptome analysis. Clean reads were aligned to the reference genome Zhenshan97B of rice (http://rice.hzau.edu.cn/rice/download_ext/ZS97RS2.LNNJ00000000.fasta.gz accessed on 20 April 2022) by HISAT [61]. HTSeq [62] was used to count the read numbers that were mapped to each gene. DESeq2 [63] was applied for differential gene expression analysis. Genes with log2 fold change ≥ 1 and q-value < 0.05 were considered to be expressed differentially. The differentially expressed genes were further analyzed with Gene Ontology and Kyoto Encyclopedia of Genes and Genomes (http://www.genome.jp/kegg/ accessed on 20 April 2022) analyses.

### 4.9. Quantitative Real-Time PCR Analysis

To validate the accuracy of RNA-seq data, qRT--PCR analyses were performed using a LightCycler FastStart DNA Master SYBR Green I kit (Roche, Basel, Switzerland) on a CFX384 Real-Time PCR detection system (C1000 TOUCH, BIO-RAD, Hercules, CA, USA).

### 4.10. Determination of Metabolite Content

The powder of the embryo, endosperm, aleurone layer, and the hull was used for metabolite extraction. A total of 100 mg dried powder was weighted and extracted overnight at 4 °C with 1.0 mL 70% aqueous methanol containing 0.1 mg L^−1^ Acycloguanosine-1 (internal standard) for lipid-solubility metabolites. A liquid chromatography–electrospray ionization–tandem mass spectrometry (LC-ESI-MS/MS) system was used for the relative quantification of metabolites in rice seeds samples. Quantification of metabolites was carried out using a scheduled multiple reaction monitoring method [64].

### 4.11. Metabolomics Analysis

MetaboAnalyst 3.0 (https://www.metaboanalyst.ca/MetaboAnalyst/home.xhtml accessed on 20 April 2022) was employed for the PCA and statistical analysis. Heatmap was performed by using pheatmap package in R (http://cran.r-project.org/web/packages/pheatmap/pheatmap.pdf accessed on 20 April 2022), while Circos plot was performed by using Circos software in perl (http://www.circos.ca/software/ accessed on 20 April 2022).

## Figures and Tables

**Table 1:** QTLs identified for four germination parameters in the NIP/ZS97 CSSL population using the single nucleotide polymorphism (SNP) bin markers.

QTLs	Chr	Intervial	Gmax			T50			T10			AUC		
			Estimate	P	PVE	Estimate	P	PVE	Estimate	P	PVE	Estimate	P	PVE
*qSST1.1*	1	38.33–38.61	−0.003	8.26 × 10^−3^	1.6%	-	-	-	1.222	2.78 × 10^−3^	1.5%	−0.44	8.82 × 10^−3^	1.5%
*qSST1.2*	1	39.05–39.95	−0.003	2.01 × 10^−3^	4.1%	-	-	-	-	-	-	−0.414	2.56 × 10^−3^	3.7%
*qSST2.1*	2	3.24–3.76	-	-	-	-	-	-	1.889	2.62 × 10^−3^	1.4%	-	-	-
*qSST2.2*	2	4.63–5.28	−0.004	1.45 × 10^−3^	3.0%	2.166	9.29 × 10^−9^	7.4%	2.808	7.25 × 10^−9^	7.1%	−0.745	1.34 × 10^−4^	3.8%
*qSST2.3*	2	6.73–9.92	−0.004	3.40 × 10^−3^	1.0%	1.216	2.31 × 10^−3^	1.7%	1.805	4.13 × 10^−5^	3.0%	−0.715	1.75 × 10^−3^	1.1%
*qSST3.1*	3	0.00–2.10	−0.005	8.54 × 10^−4^	3.0%	2.727	4.77 × 10^−14^	11.9%	3.469	8.88 × 10^−16^	11.2%	−0.783	1.70 × 10^−4^	3.6%
*qSST3.2*	3	26.20–28.32	−0.003	8.84 × 10^−5^	13.8%	0.613	7.28 × 10^−3^	2.9%	0.98	1.21 × 10^−4^	8.5%	−0.497	6.95 × 10^−5^	13.9%
*qSST3.3*	3	31.69–31.76	-	-	-	-	-	-	−1.276	8.51 × 10^−3^	0.6%	-	-	-
*qSST4.1*	4	0.00–6.49	−0.005	2.21 × 10^−3^	2.6%	-	-	-	-	-	-	−0.478	3.23 × 10^−3^	1.0%
*qSST4.2*	4	19.24–27.22	−0.003	1.69 × 10^−4^	17.2%	0.893	3.00 × 10^−5^	6.1%	1.087	2.01 × 10^−5^	10.2%	−0.432	1.90 × 10^−4^	16.7%
*qSST5.1*	5	16.92–16.97	-	-	-	-	-	-	2.065	3.61 × 10^−3^	0.7%	-	-	-
*qSST6.1*	6	3.08–6.75	-	-	-	-	-	-	2.065	3.61 × 10^−3^	2.9%	-	-	-
*qSST7.1*	7	6.82–15.23	-	-	-	1.022	9.06 × 10^−4^	4.4%	1.207	1.77 × 10^−3^	3.3%	-	-	-
*qSST9.1*	9	15.50–21.15	−0.004	1.75 × 10^−3^	4.7%	1.413	2.10 × 10^−6^	9.9%	2.314	2.63 × 10^−10^	14.5%	−0.585	2.46 × 10^−3^	4.3%
*qSST10.1*	10	9.08–9.41	0.005	3.35 × 10^−3^	1.0%	-	-	-	-	-	-	0.828	3.71 × 10^−3^	0.9%
*qSST10.2*	10	12.11–14.93	0.005	8.55 × 10^−3^	0.8%	-	-	-	-	-	-	0.857	5.25 × 10^−3^	1.6%
*Total*	-	-	-	-	52.8%	-	-	44.3%	-	-	64.9%	-	-	52.1%

The 4 indicators used to measure the germination rate after artificial aging of seeds, Gmax: maximum germination rate of 7 days germination experiment, T50: the time it takes for germinated seeds to reach 50% of germination, T10: the time it takes for germinated seeds to reach 10% of germination, AUC: area under the germination curve, Chr: Chromosome, Interval: Based on the physical position of a given bin of the Rice Genome Annotation Project (version 7), Estimate: Effect value, indicating the lowest when two or more consecutive bands are significant *p*-value, P: *p*-value, PVE: interpretation of phenotypic differences.

**Table 2:** Seed storability phenotype shown by Gmax and AUC in four NILs and ZS97.

Sample	Gmax (%)	AUC (h)
NZ19	28.2 ± 4.4 *	22.5 ± 3.8 *
NZ24	24.0 ± 1.2 **	16.9 ± 2.0 **
NZ29	32.0 ± 3.5 *	27.6 ± 2.5 *
NZ127	34.0 ± 3.1 *	23.7 ± 2.9 *
ZS97	57.3 ± 4.7	48.7 ± 4.6

*,** indicate significant difference with ZS97 at *p* < 0.05, *p* < 0.01 by Student’s *t*-test, respectively.

**Table 3:** Selected down-regulated DEGs in seven categories.

Organ	Category	Gene ID	Gene	Annotation
Embryo	Hormone	LOC_Os11g30500		HVA22 (ABA-and stress-inducible)
Embryo	Cell wall	LOC_Os02g51040		expansin precursor
Embryo	Transportation	LOC_Os07g26630	*OsPIP2;4*	aquaporin protein
Embryo	Stress	LOC_Os08g43120		Plant PDR ABC transporter-associated domain-containing protein
Endosperm	Hormone	LOC_Os01g72900	*OsASR5*	abscisic stress-ripening
Endosperm	Hormone	LOC_Os01g72910	*OsASR6*	abscisic stress-ripening
Endosperm	Hormone	LOC_Os04g44500		GEM (ABA-responsive protein-like)
Endosperm	Cell wall	LOC_Os01g71860		glycosyl hydrolases family 17
Endosperm	Energy supply	LOC_Os02g52700		alpha-amylase precursor, putative
Endosperm	Stress	LOC_Os06g45140	*OsbZIP52*	bZIP transcription factor domain-containing protein
Endosperm	Stress	LOC_Os08g08970	*OsGLP8-3*	OsGLP8-3; GER2- Cupin domain-containing protein
Endosperm	Germination	LOC_Os09g32290		FAD-dependent oxidoreductase domain-containing protein
Aleurone layer	Hormone	LOC_Os03g40540	*OsDWARF*	brassinosteroid-deficient dwarf1, cytochrome P450
Aleurone layer	Cell wall	LOC_Os07g14850	*CESA6*	CESA6-cellulose synthase
Aleurone layer	Cell wall	LOC_Os08g34790	*Os4CL5*	AMP-binding domain-containing protein
Aleurone layer	Transportation	LOC_Os04g48290		MATE efflux family protein
Embryo, Endosperm	Energy supply	LOC_Os09g28400		alpha-amylase precursor
Embryo, Endosperm	Germination	LOC_Os05g39310	*OsPDC1*	thiamine pyrophosphate enzyme, C-terminal TPP binding domain-containing protein
Embryo, Aleurone layer	Hormone	LOC_Os07g05940	*OsNCED4*	9-cis-epoxycarotenoid dioxygenase 1, chloroplast precursor, putative
Embryo, Aleurone layer	Transportation	LOC_Os01g48680	*OsTPC1*	two-pore calcium channel protein 1
Endosperm, Aleurone layer	Energy supply	LOC_Os02g52710	*RAmy1A*	RAmy1A-alpha-amylase precursor
Endosperm, Aleurone layer	Hormone	LOC_Os07g40290	*OsGH3.8*	OsGH3.8-Probable indole-3-acetic acid-amido synthetase
Embryo, Endosperm, Aleurone layer	Stress	LOC_Os03g53340	*OsHsfA2a*	OsHsfA2a-HSF-type DNA-binding domain-containing protein

**Table 4:** Information of 12 candidate genes.

Source	NIL	Tissue	Gene (MSU)	Gene Product Name
Common DEGs	NZ19, NZ24, NZ29	Endosperm	LOC_Os01g71860	glycosyl hydrolases family 17, putative
Common DEGs	NZ127	Aleurone layer	LOC_Os04g33640	glycosyl hydrolases family 17, putative
Common DEGs	NZ127	Endosperm	LOC_Os04g44500	GEM, putative
Common DEGs	NZ127	Endosperm	LOC_Os04g44580	expressed protein
Common DEGs	NZ127	Aleurone layer	LOC_Os04g48290	MATE efflux family protein, putative
Common DEGs	NZ127	Endosperm	LOC_Os04g52504	adhesive/proline-rich protein, putative
KEGG pathway	NZ127	Embryo, Aleurone layer	LOC_Os04g39864	beta-glucosidase homologue, similar to Os4Bglu12 exoglucanase/beta-glucosidase
KEGG pathway	NZ127	Endosperm	LOC_Os04g43410	Os4bglu18-monolignol beta-glucoside homologue
KEGG pathway	NZ127	Endosperm	LOC_Os04g43800	phenylalanine ammonia-lyase, putative
KEGG pathway	NZ127	Endosperm	LOC_Os04g44150	gibberellin 2-oxidase gene
KEGG pathway	NZ127	Embryo, Aleurone layer	LOC_Os04g52210	terpene synthase, putative
KEGG pathway	NZ127	Endosperm, Aleurone layer	LOC_Os04g53630	pentatricopeptide, putative
KEGG pathway	NZ19	Aleurone layer		

## Data Availability

All raw data reported in this article have been deposited in the National Center for Biotechnology Information sequence read archive (BioProject number: PRJNA833206). All data supporting this research result can be obtained in the article and within its Supplementary Materials published online. Additional data related to this article may be requested from the authors.

## Supplementary Materials

The following supporting information can be downloaded at: https://www.mdpi.com/article/10.3390/plants11121570/s1. Figure S1: KEGG pathway enrichment analysis of the DEGs in the four non-storable NILs compared with ZS97; Figure S2: qPCR validation of the selected key genes. Yellow bar represents the relative expression of the corresponding gene in ZS97, and light green bar represents the relative expression of the corresponding gene in the non-storable material NZ127. Table S1: The detailed information of the introgressed region of four selected non-storable NILs; Table S2: Common DEGs of the four selected non-storable NILs.
