# Supplementary material for: Insights into the Regulation of Rice Seed Storability by Seed Tissue-Specific Transcriptomic and Metabolic Profiling
Source: Plants (Basel). 2022 Jun 14;11(12):1570. doi: 10.3390/plants11121570 (PMC9231264; doi:10.3390/plants11121570)
Supplement: Supplementary file 1 [file plants-11-01570-s001.zip › plants-1726817-supplementary.pdf]

plants

MDPI

Table S1. The detailed information of the introgressed region of four selected non-storable NILs.

**Sample**	**18NZ19**		**18NZ24**	**18NZ29**	**18NZ127**	
**chromosome**	**1**	**1**	**1**	**1**	**4**	**4**
start	39140688	40869276	38384401	39823456	4407267	19238998
end	39951148	41866313	41866313	41866313	4410584	32147532
start-100kb	39040688	40769276	38284401	39723456	4307267	19138998
end+100kb	40051148	41966313	41966313	41966313	4510584	32247532
start-loc	LOC_Os01g67410	LOC_Os01g70380	LOC_Os01g65950	LOC_Os01g68380	LOC_Os04g08070	LOC_Os04g31960
end-loc	LOC_Os01g68760	LOC_Os01g72340	LOC_Os01g72340	LOC_Os01g72340	LOC_Os04g08400	LOC_Os04g54110
start-ZS	OsZS_01G0649000	-	OsZS_01G0634100	OsZS_01G0659900	-	OsZS_04G0290500
ZS97-location	Chr01:40,465,496..40,470,311		Chr01:39,588,680..39,592,104	Chr01:41,098,119..41,105,099		Chr04:19,571,507..19,573,709
end-ZS97	OsZS_01G0664100	OsZS_01G0691800	OsZS_01G0691800	OsZS_01G0691800	OsZS_04G0069300	OsZS_04G0502000
ZS97-location	Chr01:41,307,157..41,311,951	Chr01:43,186,880..43,195,569	Chr01:43,186,880..43,195,569	Chr01:43,186,880..43,195,569	Chr04:4,308,832..4,314,671	Chr04:33,083,532..33,089,976

Table S2. Common DEGs of the four selected non-storable NILs.

Regu-lated	Organ	Gene(ZS97 ID)	Gene(MSU)	Gene Product Name	CDS Coordinates (5'-3')
UP	embryo	OsZS_01G0619700	LOC_Os01g64640	histone H3, putative, expressed	37513746-37514564
UP	embryo	OsZS_10G0371200	LOC_Os10g39410	Core histone H2A/H2B/H3/H4 domain-containing protein, putative, expressed	21019903-21019255
UP	embryo	OsZS_03G0017200	LOC_Os03g02780	Core histone H2A/H2B/H3/H4 domain-containing protein, putative, expressed	1080711-1081442
UP	embryo	OsZS_05G0343800	LOC_Os05g36280	histone H3, putative, expressed	21500179-21499419
UP	embryo	OsZS_07G0012300	LOC_Os07g02430	expressed protein	835728-834299
UP	embryo	OsZS_07G0366800	LOC_Os07g36500	Core histone H2A/H2B/H3/H4 domain-containing protein, putative, expressed	21827213-21827867
UP	embryo	OsZS_08G0409700	LOC_Os08g38300	Core histone H2A/H2B/H3/H4 domain-containing protein, putative, expressed	24261320-24262356
UP	embryo	OsZS_03G0163900	LOC_Os03g17100	Core histone H2A/H2B/H3/H4 domain-containing protein, putative, expressed	9499826-9498900
UP	embryo	OsZS_01G0487200	LOC_Os01g51770	outer mitochondrial membrane porin, putative, expressed	29773361-29770182
UP	embryo	OsZS_02G0484100	LOC_Os02g45940	Core histone H2A/H2B/H3/H4 domain-containing protein, putative, expressed	27994741-27993898
UP	embryo	OsZS_12G0368600	LOC_Os12g40710	helix-loop-helix DNA-binding domain-containing protein, expressed	25202156-25204363

UP	embryo	OsZS_03G0030800	-	-	-
UP	embryo	OsZS_05G0012400	LOC_Os05g02300	Core histone H2A/H2B/H3/H4 domain-containing protein, putative, expressed	738204-739079
UP	embryo	OsZS_02G0424600	LOC_Os02g39960	exostosin family protein, putative, expressed	24165653-24163553
UP	embryo	OsZS_11G0025400	LOC_Os11g03430	CDC45B - Putative DNA replication initiation protein, expressed	1306316-1308723
UP	embryo	OsZS_11G0031900	LOC_Os11g04180	dual specificity protein phosphatase, putative, expressed	1699220-1696427
UP	embryo	OsZS_06G0402000	LOC_Os06g44240	gp176, putative, expressed	26690714-26692639
UP	embryo	OsZS_06G0080200	-	-	-
UP	endosperm	Novel00873	-	-	-
UP	endosperm	OsZS_01G0619700	LOC_Os01g64640	histone H3, putative, expressed	37513746-37514564
UP	endosperm	OsZS_07G0394000	LOC_Os07g39060	expressed protein	23416886-23415585
UP	endosperm	OsZS_06G0397900	-	-	-
UP	endosperm	OsZS_03G0273600	-	-	-
UP	endosperm	Novel00689	-	-	-
UP	endosperm	OsZS_05G0199000	LOC_Os05g22830	DNA-directed RNA polymerase subunit beta, putative, expressed	12953646-12957954

UP	endosperm	OsZS_03G0037900	LOC_Os03g04780	RNA recognition motif-containing protein, putative, expressed	2277298-2276860
UP	endosperm	OsZS_04G0489000	-	-	-
UP	endosperm	OsZS_10G0200900	LOC_Os10g21130	expressed protein	10751985-10748893
UP	endosperm	OsZS_03G0343400	LOC_Os03g36080	expressed protein	20019699-20018644
UP	endosperm	OsZS_06G0455300	-	-	-
UP	endosperm	OsZS_01G0588600	LOC_Os01g61510	ammonium transporter protein, putative, expressed	35588377-35585865
UP	endosperm	OsZS_08G0123000	LOC_Os08g13430	expressed protein	7993729-7991097
UP	endosperm	OsZS_03G0245600	-	-	-
UP	endosperm	OsZS_07G0076000	-	-	-
UP	aleurone layer	Novel00689	-	-	-
UP	aleurone layer	OsZS_01G0619700	LOC_Os01g64640	histone H3, putative, expressed	37513746-37514564
UP	aleurone layer	OsZS_03G0597100	-	-	-
UP	aleurone layer	OsZS_03G0017200	LOC_Os03g02780	Core histone H2A/H2B/H3/H4 domain-containing protein, putative, expressed	1080711-1081442

UP	aleurone layer	OsZS_12G0199400	-	-	-
UP	aleurone layer	OsZS_04G0256000	-	-	-
UP	aleurone layer	OsZS_08G0409700	LOC_Os08g38300	Core histone H2A/H2B/H3/H4 domain-containing protein, putative, expressed	24261320-24262356
UP	aleurone layer	OsZS_03G0343400	LOC_Os03g36080	expressed protein	20019699-20018644
UP	aleurone layer	OsZS_11G0092900	LOC_Os11g10470	expressed protein	5711249-5710546
UP	aleurone layer	OsZS_04G0458000	LOC_Os04g49420	Core histone H2A/H2B/H3/H4 domain-containing protein, putative, expressed	29489558-29487977
UP	aleurone layer	OsZS_01G0107800	LOC_Os01g12020	LTPL18 - Protease inhibitor/seed storage/LTP family protein precursor, expressed	6541005-6543076
UP	aleurone layer	OsZS_09G0322200	-	-	-
DOWN	embryo	OsZS_10G0210800	-	-	-
DOWN	embryo	OsZS_06G0037400	LOC_Os06g04990	early nodulin 93 ENOD93 protein, putative, expressed	2200544-2199242
DOWN	embryo	OsZS_11G0293200	LOC_Os11g30500	HVA22, putative, expressed	17729025-17730347
DOWN	embryo	OsZS_05G0369600	LOC_Os05g39310	thiamine pyrophosphate enzyme, C-terminal TPP binding domain-containing protein, expressed	23058937-23061264
DOWN	embryo	OsZS_03G0524900	LOC_Os03g53340	OsHsfA2a-HSF-type DNA-binding domain-containing protein, expressed	30607159-30604067

DOWN	embryo	OsZS_02G0541600	LOC_Os02g51040	expansin precursor, putative, expressed	31220568-31222229
DOWN	embryo	OsZS_03G0445900	LOC_Os03g45250	2-aminoethanethiol dioxygenase, putative, expressed	25547180-25549126
DOWN	embryo	OsZS_10G0349800	LOC_Os10g37190	protein kinase domain-containing protein, expressed	19900928-19899083
DOWN	embryo	OsZS_02G0113600	LOC_Os02g12480	expressed protein	6513827-6508832
DOWN	embryo	OsZS_12G0290500	LOC_Os12g32390	expressed protein	19543820-19544475
DOWN	embryo	OsZS_01G0456000	LOC_Os01g48680	two pore calcium channel protein 1, putative, expressed	27920980-27906608
DOWN	embryo	OsZS_07G0373000	LOC_Os07g37210	MYB family transcription factor, putative, expressed	22295309-22293735
DOWN	embryo	OsZS_10G0400100	-	-	-
DOWN	embryo	OsZS_10G0091600	LOC_Os10g08790	expressed protein	4762869-4763333
DOWN	embryo	OsZS_12G0166000	-	-	-
DOWN	embryo	OsZS_02G0033800	LOC_Os02g04369	expressed protein	1928625-1929506
DOWN	embryo	OsZS_06G0375900	LOC_Os06g41880	OsSub51 - Putative Subtilisin homologue, expressed	25118149-25126142
DOWN	embryo	OsZS_07G0442800	LOC_Os07g44180	OsRCI2-10 - Hydrophobic protein LTI6A, expressed	26403674-26404729

DOWN	embryo	OsZS_04G0511400	LOC_Os04g55100	expressed protein	32764819-32766366
DOWN	embryo	OsZS_04G0157400	LOC_Os04g17660	rhodanese-like domain-containing protein, putative, expressed	9674123-9671230
DOWN	embryo	OsZS_01G0562200	LOC_Os01g58850	circadian clock coupling factor-related, putative, expressed	33997100-33998320
DOWN	embryo	OsZS_09G0253400	LOC_Os09g27010	tyrosine protein kinase domain-containing protein, putative, expressed	16425218-16422278
DOWN	embryo	OsZS_03G0180000	LOC_Os03g18770	wound-induced protein WI12, putative, expressed	10518361-10517108
DOWN	embryo	OsZS_10G0381900	LOC_Os10g40440	LTPL140 - Protease inhibitor/seed storage/LTP family protein precursor, expressed	21663224-21662437
DOWN	embryo	OsZS_06G0066500	LOC_Os06g07914	hydroxylase, putative, expressed	3846106-3848325
DOWN	embryo	OsZS_03G0578500	LOC_Os03g59110	pheophorbide a oxygenase, chloroplast precursor, putative, expressed	33649350-33653755
DOWN	embryo	OsZS_03G0424800	LOC_Os03g42710	WD-40 repeat family protein, putative, expressed	23781111-23782726
DOWN	embryo	OsZS_02G0054000	LOC_Os02g06090	phytosulfokine receptor precursor, putative, expressed	3037225-3040483
DOWN	embryo	OsZS_02G0600000	-	-	-
DOWN	embryo	OsZS_11G0304200	LOC_Os11g31540	BRASSINOSTEROID INSENSITIVE 1-associated receptor kinase 1 precursor, putative, expressed	18432558-18430460
DOWN	embryo	OsZS_09G0072200	LOC_Os09g08470	retrotransposon protein, putative, unclassified, expressed	4426072-4420207

DOWN	embryo	OsZS_09G0278900	LOC_Os09g29100	cyclin, putative, expressed	17680696-17683508
DOWN	embryo	OsZS_08G0456300	LOC_Os08g43120	Plant PDR ABC transporter associated domain-containing protein, expressed	27268083-27277540
DOWN	embryo	OsZS_01G0480600	LOC_Os01g51140	helix-loop-helix DNA-binding domain-containing protein, expressed	29385646-29382708
DOWN	embryo	OsZS_03G0544700	LOC_Os03g55880	expressed protein	31824273-31824695
DOWN	embryo	OsZS_08G0442300	LOC_Os08g41520	zinc finger, C3HC4 type domain-containing protein, expressed	26232707-26231788
DOWN	embryo	OsZS_06G0098500	LOC_Os06g11150	DUF1645 domain-containing protein, putative, expressed	5847981-5849957
DOWN	embryo	OsZS_03G0085800	LOC_Os03g10100	transporter family protein, putative, expressed	5112102-5114453
DOWN	embryo	Novel01060	-	-	-
DOWN	embryo	OsZS_01G0688100	LOC_Os01g71830	glycosyl hydrolases family 17, putative, expressed	41603297-41604680
DOWN	embryo	OsZS_02G0454300	LOC_Os02g43280	aldehyde dehydrogenase, putative, expressed	26079797-26085572
DOWN	embryo	Novel00619	-	-	-
DOWN	embryo	OsZS_07G0478900	LOC_Os07g48050	peroxidase precursor, putative, expressed	28688443-28690373
DOWN	embryo	OsZS_04G0504000	LOC_Os04g54310	expressed protein	32337872-32336347
DOWN	embryo	OsZS_11G0292100	LOC_Os11g30360	expressed protein	17625788-17626349

DOWN	embryo	OsZS_07G0259200	LOC_Os07g26630	aquaporin protein, putative, expressed	15358960-15360495
DOWN	embryo	OsZS_08G0455200	LOC_Os08g42990	expressed protein	27171307-27169279
DOWN	embryo	OsZS_05G0408100	LOC_Os05g43910	cytochrome P450, putative, expressed	25543222-25546410
DOWN	embryo	OsZS_04G0522000	LOC_Os04g56180	peroxidase precursor, putative, expressed	33506116-33499949
DOWN	embryo	OsZS_11G0074200	-	-	-
DOWN	embryo	OsZS_09G0270200	LOC_Os09g28400	alpha-amylase precursor, putative, expressed	17288993-17291295
DOWN	embryo	OsZS_02G0421000	LOC_Os02g39640	expressed protein	23914443-23916210
DOWN	embryo	OsZS_06G0006300	LOC_Os06g01810	expressed protein	468425-465680
DOWN	embryo	OsZS_07G0478800	LOC_Os07g47990	peroxidase precursor, putative, expressed	28660188-28661910
DOWN	embryo	OsZS_07G0478800	LOC_Os07g48010	peroxidase precursor, putative, expressed	28669498-28671387
DOWN	embryo	OsZS_11G0060800	LOC_Os11g07060	receptor protein kinase CLAVATA1 precursor, putative, expressed	3517792-3522211
DOWN	embryo	OsZS_12G0206200	LOC_Os12g23280	heavy metal-associated domain-containing protein, expressed	13177329-13179007
DOWN	embryo	OsZS_09G0351800	LOC_Os09g38320	phytoene synthase, chloroplast precursor, putative, expressed	22045393-22041967

DOWN	embryo	OsZS_06G0029100	LOC_Os06g04220	expressed protein	1780472-1781052
DOWN	embryo	OsZS_08G0009600	-	-	-
DOWN	embryo	Novel00513	-	-	-
DOWN	embryo	OsZS_07G0308700	LOC_Os07g30510	TKL_IRAK_DUF26-la.3 - DUF26 kinases have homology to DUF26 containing loci, expressed	18051110-18047057
DOWN	embryo	OsZS_07G0051300	LOC_Os07g05940	9-cis-epoxycarotenoid dioxygenase 1, chloroplast precursor, putative, expressed	2870686-2872832
DOWN	embryo	OsZS_10G0129500	LOC_Os10g13700	phosphoenolpyruvate carboxykinase, putative, expressed	7444108-7438335
DOWN	embryo	OsZS_08G0434500	LOC_Os08g40690	glycosyl hydrolase, putative, expressed	25762457-25763824
DOWN	embryo	OsZS_06G0413200	-	-	-
DOWN	embryo	OsZS_07G0233900	LOC_Os07g23410	fatty acid desaturase, putative, expressed	13202543-13201060
DOWN	embryo	OsZS_06G0365100	LOC_Os06g40870	expressed protein	24375212-24373213
DOWN	embryo	OsZS_02G0529700	-	-	-
DOWN	embryo	OsZS_03G0338700	LOC_Os03g35640	retrotransposon protein, putative, unclassified, expressed	19743648-19740433
DOWN	embryo	OsZS_12G0382900	LOC_Os12g42220	expressed protein	26195982-26194509
DOWN	embryo	OsZS_08G0075000	-	-	-

DOWN	embryo	OsZS_02G0517500	LOC_Os02g48900	aspartic proteinase nepenthesin-1 precursor, putative, expressed	29911310-29916317
DOWN	embryo	OsZS_09G0294800	LOC_Os09g31000	EF hand family protein, expressed	18662882-18663721
DOWN	embryo	OsZS_09G0146800	-	-	-
DOWN	embryo	OsZS_03G0153600	LOC_Os03g16260	protein kinase, putative, expressed	8966806-8963965
DOWN	embryo	Novel00537	-	-	-
DOWN	embryo	OsZS_11G0429400	LOC_Os11g44800	expressed protein	27111943-27113472
DOWN	embryo	OsZS_11G0374800	LOC_Os11g39370	BRASSINOSTEROID INSENSITIVE 1-associated receptor kinase 1 precursor, putative, expressed	23436807-23431233
DOWN	embryo	OsZS_02G0149300	LOC_Os02g15340	no apical meristem protein, putative, expressed	8582543-8584541
DOWN	embryo	OsZS_09G0141400	LOC_Os09g15660	expressed protein	9556218-9554911
DOWN	embryo	OsZS_02G0432200	LOC_Os02g40700	enzyme of the cupin superfamily protein, putative, expressed	24676363-24677364
DOWN	embryo	OsZS_01G0193200	LOC_Os01g21034	pectinesterase, putative, expressed	11741384-11750709
DOWN	embryo	OsZS_06G0012300	-	-	-
DOWN	embryo	OsZS_12G0091900	LOC_Os12g10710	NB-ARC domain-containing protein, expressed	5752994-5746099
DOWN	embryo	OsZS_09G0330700	LOC_Os09g36250	MYB family transcription factor, putative, expressed	20919808-20915301

DOWN	embryo	OsZS_01G0038000	LOC_Os01g04690	expressed protein	2109635-2108947
DOWN	embryo	OsZS_04G0408800	LOC_Os04g45020	MYB family transcription factor, putative, expressed	26637819-26640662
DOWN	embryo	OsZS_07G0402700	LOC_Os07g39950	mitotic checkpoint protein, putative, expressed	23965883-23970059
DOWN	embryo	OsZS_07G0402700	LOC_Os07g39960	ZOS7-07 - C2H2 zinc finger protein, expressed	23973458-23976719
DOWN	embryo	OsZS_01G0435100	-	-	-
DOWN	embryo	OsZS_06G0324900	LOC_Os06g37500	cytokinin dehydrogenase precursor, putative, expressed	22194963-22192033
DOWN	embryo	OsZS_04G0347900	-	-	-
DOWN	embryo	OsZS_06G0143700	-	-	-
DOWN	embryo	OsZS_05G0249600	LOC_Os05g27510	expressed protein	16001765-16002728
DOWN	embryo	OsZS_10G0348100	LOC_Os10g36924	aquaporin protein, putative, expressed	19785158-19778055
DOWN	embryo	OsZS_03G0477900	-	-	-
DOWN	embryo	OsZS_09G0321100	-	-	-
DOWN	embryo	OsZS_07G0237900	LOC_Os07g23944	glycosyl hydrolase, family 31, putative, expressed	13534771-13548807
DOWN	embryo	Novel01032	-	-	-

DOWN	embryo	OsZS_10G0214500	LOC_Os10g22520	cellulase, putative, expressed	11673599-11666779
DOWN	embryo	OsZS_02G0446300	LOC_Os02g42620	protein kinase, putative, expressed	25641155-25639087
DOWN	embryo	OsZS_02G0150400	LOC_Os02g15540	expressed protein	8715055-8714095
DOWN	embryo	OsZS_12G0377000	-	-	-
DOWN	embryo	OsZS_01G0677200	-	-	-
DOWN	embryo	OsZS_12G0377100	-	-	-
DOWN	embryo	OsZS_01G0426700	LOC_Os01g45550	auxin efflux carrier component, putative, expressed	25863702-25868361
DOWN	embryo	OsZS_02G0408100	LOC_Os02g38260	glycosyl hydrolase family 5 protein, putative, expressed	23142015-23144272
DOWN	embryo	OsZS_12G0031400	LOC_Os12g04080	transferase family protein, putative, expressed	1714665-1713136
DOWN	embryo	OsZS_02G0560200	-	-	-
DOWN	embryo	OsZS_09G0143500	LOC_Os09g15840	NBS-LRR disease resistance protein, putative, expressed	9674695-9674000
DOWN	embryo	OsZS_12G0039600	-	-	-
DOWN	embryo	OsZS_01G0325600	LOC_Os01g34560	3-ketoacyl-CoA synthase, putative, expressed	19059233-19061180

DOWN	embryo	OsZS_10G0124700	LOC_Os10g12400	nodulin, putative, expressed	6903298-6906138
DOWN	embryo	OsZS_06G0145900	-	-	-
DOWN	embryo	OsZS_02G0421200	LOC_Os02g39660	receptor kinase, putative, expressed	23952926-23954617
DOWN	embryo	OsZS_02G0375500	LOC_Os02g35190	chloride channel protein, putative, expressed	21161370-21150702
DOWN	embryo	OsZS_10G0206300	-	-	-
DOWN	embryo	OsZS_02G0386000	LOC_Os02g36190	cytochrome P450, putative, expressed	21813460-21810334
DOWN	embryo	Novel01459	-	-	-
DOWN	embryo	OsZS_06G0417200	LOC_Os06g45610	expressed protein	27614006-27614830
DOWN	embryo	OsZS_04G0106900	-	-	-
DOWN	embryo	Novel01216	-	-	-
DOWN	endosperm	OsZS_03G0592700	LOC_Os03g60720	expansin precursor, putative, expressed	34511279-34508262
DOWN	endosperm	OsZS_09G0270200	LOC_Os09g28400	alpha-amylase precursor, putative, expressed	17288993-17291295
DOWN	endosperm	OsZS_01G0688400	LOC_Os01g71860	glycosyl hydrolases family 17, putative, expressed	41629299-41631914
DOWN	endosperm	OsZS_02G0558200	LOC_Os02g52710	RAmy1A-alpha-amylase precursor, putative, expressed	32248279-32250180

DOWN	endosperm	OsZS_01G0697<br>500	LOC_Os01g72<br>910	abscisic stress-ripening, putative, expressed	42282832-42283632
DOWN	endosperm	OsZS_01G0697<br>400	LOC_Os01g72<br>900	abscisic stress-ripening, putative, expressed	42281973-42281272
DOWN	endosperm	OsZS_01G0079<br>900	LOC_Os01g09<br>220	transposon protein, putative, CACTA, En/Spm sub-class, expressed	4646427-4648260
DOWN	endosperm	OsZS_02G0558<br>100	LOC_Os02g52<br>700	alpha-amylase precursor, putative, expressed	32243146-32245056
DOWN	endosperm	OsZS_04G0486<br>200	LOC_Os04g52<br>504	adhesive/proline-rich protein, putative, expressed	31221018-31219819
DOWN	endosperm	OsZS_03G0524<br>900	LOC_Os03g53<br>340	OsHsfA2a-HSF-type DNA-binding domain-containing protein, expressed	30607159-30604067
DOWN	endosperm	OsZS_03G0110<br>600	LOC_Os03g12<br>510	non-symbiotic hemoglobin 2, putative, expressed	6626083-6624958
DOWN	endosperm	OsZS_02G0388<br>200	LOC_Os02g36<br>350	expressed protein	21951775-21949884
DOWN	endosperm	OsZS_02G0113<br>600	LOC_Os02g12<br>480	expressed protein	6513827-6508832
DOWN	endosperm	OsZS_09G0304<br>800	LOC_Os09g32<br>290	FAD dependent oxidoreductase domain-containing protein, expressed	19275775-19272307
DOWN	endosperm	OsZS_07G0490<br>700	LOC_Os07g49<br>120	sex determination protein tasselseed-2, putative, expressed	29419191-29417888
DOWN	endosperm	OsZS_06G0413<br>000	LOC_Os06g45<br>140	bZIP transcription factor domain-containing protein, expressed	27302107-27299914
DOWN	endosperm	OsZS_01G0562<br>200	LOC_Os01g58<br>850	circadian clock coupling factor-related, putative, expressed	33997100-33998320

DOWN	endosperm	OsZS_09G0072200	LOC_Os09g08470	retrotransposon protein, putative, unclassified, expressed	4426072-4420207
DOWN	endosperm	Novel01602	-	-	-
DOWN	endosperm	OsZS_06G0231700	-	-	-
DOWN	endosperm	OsZS_04G0404500	LOC_Os04g44580	expressed protein	26385985-26381989
DOWN	endosperm	OsZS_07G0407300	LOC_Os07g40290	OsGH3.8 - Probable indole-3-acetic acid-amido synthetase, expressed	24152079-24149649
DOWN	endosperm	OsZS_08G0082000	LOC_Os08g08970	OsGLP8-3; GER2	5222214-5223311
DOWN	endosperm	OsZS_01G0450000	LOC_Os01g48120	expressed protein	27548239-27546819
DOWN	endosperm	OsZS_03G0372600	LOC_Os03g38540	folic acid binding protein, putative, expressed	21392896-21395039
DOWN	endosperm	OsZS_02G0267300	LOC_Os02g25860	glutelin, putative, expressed	15151364-15153319
DOWN	endosperm	OsZS_04G0532200	LOC_Os04g57200	heavy metal transport/detoxification protein, putative, expressed	34080603-34081927
DOWN	endosperm	OsZS_09G0052200	-	-	-
DOWN	endosperm	OsZS_03G0076900	LOC_Os03g08930	helix-loop-helix DNA-binding protein, putative, expressed	4628935-4630514
DOWN	endosperm	OsZS_05G0014600	LOC_Os05g02540	F-box protein interaction domain-containing protein, expressed	883703-884602
DOWN	endosperm	OsZS_03G0068600	LOC_Os03g08250	expressed protein	4200735-4199809

DOWN	endosperm	OsZS_02G0267200	LOC_Os02g25860	glutelin, putative, expressed	15151364-15153319
DOWN	endosperm	OsZS_04G0379300	-	-	-
DOWN	endosperm	OsZS_04G0522700	LOC_Os04g56240	lipase, putative, expressed	33530075-33533747
DOWN	endosperm	OsZS_01G0535900	-	-	-
DOWN	endosperm	OsZS_04G0403700	LOC_Os04g44500	GEM, putative, expressed	26343781-26342443
DOWN	endosperm	OsZS_01G0049400	LOC_Os01g05840	oxidoreductase, short chain dehydrogenase/reductase family domain-containing family, expressed	2800436-2803742
DOWN	endosperm	Novel00240	-	-	-
DOWN	endosperm	OsZS_10G0400100	-	-	-
DOWN	endosperm	OsZS_05G0059200	LOC_Os05g07720	alliin lyase precursor, putative, expressed	4152905-4155895
DOWN	endosperm	OsZS_03G0102400	LOC_Os03g11614	OsMADS1 - MADS-box family gene with MIKCc type-box, expressed	6052750-6061369
DOWN	endosperm	OsZS_01G0600700	-	-	-
DOWN	endosperm	OsZS_01G0427800	-	-	-
DOWN	endosperm	Novel01323	-	-	-
DOWN	endosperm	OsZS_03G0543200	-	-	-

DOWN	endosperm	OsZS_10G0269 100	LOC_Os10g28 630	homocysteine S-methyltransferase protein, putative, expressed	14921923-14925034
DOWN	endosperm	OsZS_11G0431 400	LOC_Os11g44 950	glycosyl hydrolase family 3 protein, putative, expressed	27210578-27203545
DOWN	endosperm	OsZS_05G0321 800	LOC_Os05g34 310	no apical meristem protein, putative, expressed	20310242-20308342
DOWN	endosperm	OsZS_04G0455 800	LOC_Os04g49 160	zinc finger, C3HC4 type domain-containing protein, expressed	29318456-29317203
DOWN	endosperm	OsZS_01G0688 100	LOC_Os01g71 830	glycosyl hydrolases family 17, putative, expressed	41603297-41604680
DOWN	endosperm	OsZS_02G0559 300	-	-	-
DOWN	endosperm	OsZS_09G0221 700	LOC_Os09g24 290	agenet domain-containing protein, putative, expressed	14434371-14440386
DOWN	endosperm	OsZS_05G0071 300	LOC_Os05g08 430	boron transporter protein, putative, expressed	4607413-4600979
DOWN	endosperm	OsZS_02G0191 500	LOC_Os02g19 420	expressed protein	11346778-11339959
DOWN	endosperm	OsZS_04G0403 800	LOC_Os04g44 510	GEM, putative, expressed	26348503-26347311
DOWN	endosperm	OsZS_03G0508 300	LOC_Os03g51 690	Homeobox domain-containing protein, expressed	29606861-29596277
DOWN	endosperm	OsZS_02G0418 700	LOC_Os02g39 490	carbonyl reductase 3, putative, expressed	23834725-23829190
DOWN	endosperm	OsZS_01G0593 200	LOC_Os01g62 000	pectate lyase 4 precursor, putative, expressed	35889803-35891365

DOWN	endosperm	OsZS_08G0087100	LOC_Os08g09610	expressed protein	5564424-5563233
DOWN	endosperm	OsZS_11G0300400	LOC_Os11g31090	transferase family protein, putative, expressed	18092965-18095638
DOWN	endosperm	OsZS_02G0590400	-	-	-
DOWN	endosperm	OsZS_05G0039100	LOC_Os05g05290	expressed protein	2606264-2605365
DOWN	endosperm	OsZS_11G0008000	-	-	-
DOWN	endosperm	OsZS_12G0324400	LOC_Os12g36220	inhibitor I family protein, putative, expressed	22198462-22197918
DOWN	endosperm	OsZS_10G0203300	LOC_Os10g21540	DUF677 domain-containing protein, putative, expressed	11026550-11029493
DOWN	endosperm	Novel01588	-	-	-
DOWN	endosperm	OsZS_03G0571400	LOC_Os03g58350	OsIAA14 - Auxin-responsive Aux/IAA gene family member, expressed	33247318-33246011
DOWN	endosperm	OsZS_04G0403600	-	-	-
DOWN	endosperm	OsZS_09G0312400	LOC_Os09g33555	expressed protein	19788719-19789621
DOWN	endosperm	OsZS_10G0304600	LOC_Os10g32658	retrotransposon protein, putative, Ty3-gypsy subclass, expressed	17106563-17105427
DOWN	endosperm	OsZS_02G0146200	LOC_Os02g15000	zinc finger, C3HC4 type domain-containing protein, expressed	8368328-8367222
DOWN	endosperm	OsZS_04G0511400	LOC_Os04g55100	expressed protein	32764819-32766366

DOWN	endosperm	OsZS_01G0019900	LOC_Os01g03130	expressed protein	1218122-1217006
DOWN	endosperm	OsZS_01G0456200	LOC_Os01g48700	transcription factor Dp, putative, expressed	27928433-27924901
DOWN	endosperm	OsZS_01G0411100	LOC_Os01g43610	DUF623 domain-containing protein, expressed	24982666-24981173
DOWN	endosperm	OsZS_09G0157700	LOC_Os09g17190	OsFBX320 - F-box domain-containing protein, expressed	10547371-10545687
DOWN	endosperm	OsZS_10G0391700	LOC_Os10g41330	AP2 domain-containing protein, expressed	22213115-22214678
DOWN	endosperm	OsZS_04G0373800	-	-	-
DOWN	endosperm	OsZS_01G0138400	LOC_Os01g14850	MFS18 protein precursor, putative, expressed	8306555-8305547
DOWN	endosperm	OsZS_08G0183500	LOC_Os08g19650	homeobox protein knotted-1, putative, expressed	11758701-11765266
DOWN	endosperm	OsZS_10G0381100	LOC_Os10g40324	expressed protein	21601595-21613523
DOWN	endosperm	OsZS_08G0104200	LOC_Os08g11500	expressed protein	6747622-6745844
DOWN	endosperm	OsZS_10G0052700	LOC_Os10g05660	thaumatin, putative, expressed	2829151-2830798
DOWN	endosperm	OsZS_02G0085000	LOC_Os02g09400	cytochrome P450, putative, expressed	4826405-4828225
DOWN	endosperm	OsZS_08G0156100	LOC_Os08g16660	aspartic proteinase nepenthesin precursor, putative, expressed	10193410-10189845

DOWN	aleurone layer	OsZS_05G0369600	LOC_Os05g39310	thiamine pyrophosphate enzyme, C-terminal TPP binding domain-containing protein, expressed	23058937-23061264
DOWN	aleurone layer	OsZS_07G0373000	LOC_Os07g37210	MYB family transcription factor, putative, expressed	22295309-22293735
DOWN	aleurone layer	OsZS_02G0558200	LOC_Os02g52710	RAmy1A-alpha-amylase precursor, putative, expressed	32248279-32250180
DOWN	aleurone layer	OsZS_03G0592700	LOC_Os03g60720	expansin precursor, putative, expressed	34511279-34508262
DOWN	aleurone layer	OsZS_10G0400100	-	-	-
DOWN	aleurone layer	OsZS_03G0395300	LOC_Os03g40540	cytochrome P450, putative, expressed	22538341-22542520
DOWN	aleurone layer	OsZS_02G0113600	LOC_Os02g12480	expressed protein	6513827-6508832
DOWN	aleurone layer	OsZS_02G0445900	LOC_Os02g42585	AP2 domain-containing protein, expressed	25597607-25595412
DOWN	aleurone layer	OsZS_01G0562200	LOC_Os01g58850	circadian clock coupling factor-related, putative, expressed	33997100-33998320
DOWN	aleurone layer	OsZS_06G0037400	LOC_Os06g04990	early nodulin 93 ENOD93 protein, putative, expressed	2200544-2199242
DOWN	aleurone layer	OsZS_02G0388200	LOC_Os02g36350	expressed protein	21951775-21949884
DOWN	aleurone layer	OsZS_04G0307700	LOC_Os04g33640	glycosyl hydrolases family 17, putative, expressed	20372913-20377959
DOWN	aleurone layer	OsZS_12G0206200	LOC_Os12g23280	heavy metal-associated domain-containing protein, expressed	13177329-13179007

DOWN	aleurone layer	OsZS_08G0370100	LOC_Os08g34790	AMP-binding domain-containing protein, expressed	21875498-21872411
DOWN	aleurone layer	OsZS_03G0524900	LOC_Os03g53340	OsHsfA2a-HSF-type DNA-binding domain-containing protein, expressed	30607159-30604067
DOWN	aleurone layer	OsZS_05G0369700	LOC_Os05g39320	thiamine pyrophosphate enzyme, C-terminal TPP binding domain-containing protein, expressed	23065607-23067936
DOWN	aleurone layer	OsZS_06G0424400	LOC_Os06g46340	glycosyl hydrolase, family 31, putative, expressed	28100801-28106083
DOWN	aleurone layer	OsZS_01G0279000	-	-	-
DOWN	aleurone layer	OsZS_10G0144900	-	-	-
DOWN	aleurone layer	OsZS_07G0407300	LOC_Os07g40290	OsGH3.8 - Probable indole-3-acetic acid-amido synthetase, expressed	24152079-24149649
DOWN	aleurone layer	OsZS_10G0206200	LOC_Os10g21670	dehydration stress-induced protein, putative, expressed	11110251-11111110
DOWN	aleurone layer	OsZS_12G0072700	LOC_Os12g08090	amino acid transporter, putative, expressed	4120926-4123058
DOWN	aleurone layer	OsZS_07G0051300	LOC_Os07g05940	9-cis-epoxycarotenoid dioxygenase 1, chloroplast precursor, putative, expressed	2870686-2872832
DOWN	aleurone layer	OsZS_01G0677500	-	-	-
DOWN	aleurone layer	OsZS_10G0144800	-	-	-
DOWN	aleurone layer	OsZS_02G0600000	-	-	-

DOWN	aleurone layer	OsZS_11G0120900	LOC_Os11g13810	non-lysosomal glucosylceramidase, putative, expressed	7595274-7606870
DOWN	aleurone layer	OsZS_08G0326100	LOC_Os08g30900	YDG/SRA domain-containing protein, expressed	19070540-19076379
DOWN	aleurone layer	OsZS_10G0244500	LOC_Os10g25487	NBS-LRR disease resistance protein, putative, expressed	13171237-13183587
DOWN	aleurone layer	OsZS_03G0053600	LOC_Os03g06654	flavin monooxygenase, putative, expressed	3355041-3360485
DOWN	aleurone layer	OsZS_01G0025900	LOC_Os01g03750	expressed protein	1570939-1569585
DOWN	aleurone layer	OsZS_08G0434500	LOC_Os08g40690	glycosyl hydrolase, putative, expressed	25762457-25763824
DOWN	aleurone layer	OsZS_03G0462200	LOC_Os03g47070	RXW8, putative, expressed	26623597-26626861
DOWN	aleurone layer	OsZS_01G0456000	LOC_Os01g48680	two pore calcium channel protein 1, putative, expressed	27920980-27906608
DOWN	aleurone layer	OsZS_07G0151500	LOC_Os07g14850	CESA6 - cellulose synthase, expressed	8496189-8501906
DOWN	aleurone layer	OsZS_03G0110600	LOC_Os03g12510	non-symbiotic hemoglobin 2, putative, expressed	6626083-6624958
DOWN	aleurone layer	OsZS_06G0037300	LOC_Os06g04950	early nodulin 93 ENOD93 protein, putative, expressed	2181116-2180198
DOWN	aleurone layer	OsZS_06G0413200	-	-	-
DOWN	aleurone layer	OsZS_04G0511400	LOC_Os04g55100	expressed protein	32764819-32766366

DOWN	aleurone layer	OsZS_06G0059600	LOC_Os06g07220	LTPL128 - Protease inhibitor/seed storage/LTP family protein precursor, expressed	3455733-3456811
DOWN	aleurone layer	OsZS_09G0312400	LOC_Os09g33555	expressed protein	19788719-19789621
DOWN	aleurone layer	OsZS_08G0461800	LOC_Os08g43680	glutathione S-transferase, C-terminal domain-containing protein, expressed	27609346-27614931
DOWN	aleurone layer	OsZS_12G0034500	LOC_Os12g04360	calmodulin-like protein 1, putative, expressed	1851398-1852196
DOWN	aleurone layer	OsZS_06G0035500	-	-	-
DOWN	aleurone layer	OsZS_09G0072200	LOC_Os09g08470	retrotransposon protein, putative, unclassified, expressed	4426072-4420207
DOWN	aleurone layer	OsZS_04G0201000	LOC_Os04g50830	retrotransposon protein, putative, LINE subclass, expressed	30069863-30066294
DOWN	aleurone layer	OsZS_05G0144300	LOC_Os05g16740	SHR5-receptor-like kinase, putative, expressed	9517479-9527704
DOWN	aleurone layer	OsZS_01G0113300	LOC_Os01g12560	3-methyl-2-oxobutanoate hydroxymethyltransferase, putative, expressed	6912131-6909691
DOWN	aleurone layer	OsZS_08G0349200	LOC_Os08g32880	disease resistance protein RPM1, putative, expressed	20389564-20392287
DOWN	aleurone layer	OsZS_04G0552300	LOC_Os04g59190	peroxidase precursor, putative, expressed	35208653-35207044
DOWN	aleurone layer	OsZS_06G0067100	LOC_Os06g08090	heparanase-like protein precursor, putative, expressed	3927916-3924681
DOWN	aleurone layer	OsZS_07G0251200	LOC_Os07g25800	OsFBDUF37 - F-box and DUF domain-containing protein, expressed	14820650-14822248

DOWN	aleurone layer	OsZS_12G0290500	LOC_Os12g32390	expressed protein	19543820-19544475
DOWN	aleurone layer	OsZS_07G0058000	-	-	-
DOWN	aleurone layer	OsZS_02G0332700	LOC_Os02g31920	expressed protein	18859234-18860105
DOWN	aleurone layer	Novel00513	-	-	-
DOWN	aleurone layer	OsZS_08G0474600	LOC_Os08g45180	kinase, pfkB family, putative, expressed	28370568-28375965
DOWN	aleurone layer	OsZS_06G0471600	-	-	-
DOWN	aleurone layer	OsZS_09G0193700	-	-	-
DOWN	aleurone layer	OsZS_04G0444400	LOC_Os04g48290	MATE efflux family protein, putative, expressed	28772448-28776387
DOWN	aleurone layer	OsZS_08G0322200	LOC_Os08g30520	plant protein of unknown function domain-containing protein, expressed	18776758-18778781
DOWN	aleurone layer	OsZS_02G0264000	LOC_Os02g25680	expressed protein	15034495-15033342
DOWN	aleurone layer	OsZS_09G0260600	LOC_Os09g27650	ZOS9-14 - C2H2 zinc finger protein, expressed	16822234-16825686
DOWN	aleurone layer	OsZS_09G0268800	LOC_Os09g28300	remorin C-terminal domain-containing protein, putative, expressed	17182867-17188378
DOWN	aleurone layer	OsZS_05G0135200	LOC_Os05g15880	glycosyl hydrolase, putative, expressed	8959415-8960578

DOWN	aleurone layer	OsZS_12G0351 000	LOC_Os12g39 120	protein phosphatase 2C, putative, expressed	24066293-24069860
DOWN	aleurone layer	OsZS_01G0025 800	LOC_Os01g03 730	nuclease PA3, putative, expressed	1566138-1569472
DOWN	aleurone layer	OsZS_12G0340 500	-	-	-
DOWN	aleurone layer	OsZS_08G0027 700	LOC_Os08g03 670	galactosyltransferase family protein, putative, expressed	1744492-1749163
DOWN	aleurone layer	OsZS_06G0124 000	-	-	-
DOWN	aleurone layer	OsZS_07G0480 400	LOC_Os07g48 130	potassium transporter, putative, expressed	28747059-28741520
DOWN	aleurone layer	OsZS_02G0035 400	LOC_Os02g04 540	retrotransposon protein, putative, unclassified, expressed	2023488-2020969
DOWN	aleurone layer	Novel00049	-	-	-

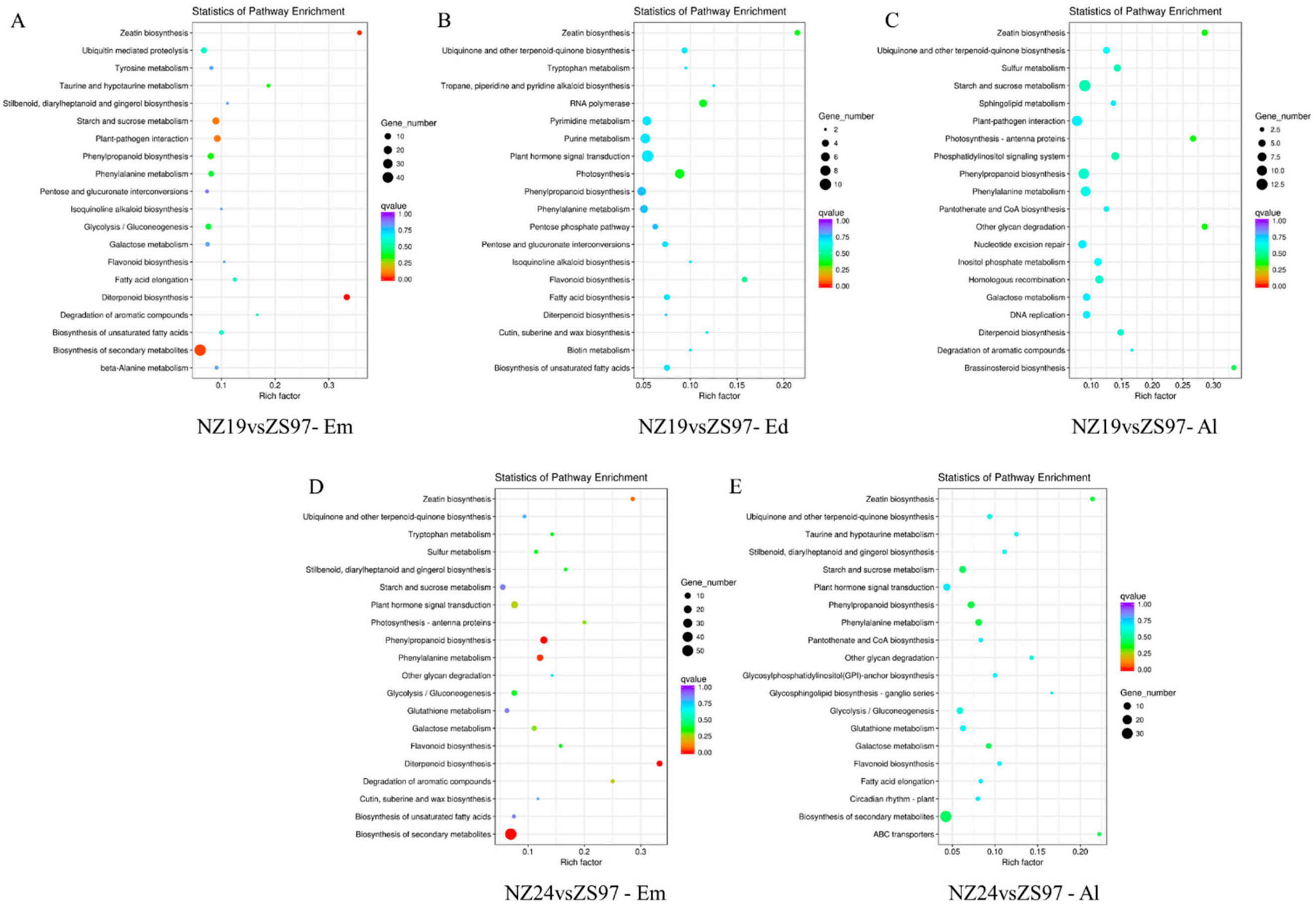

A
Statistics of Pathway Enrichment
Zeatin biosynthesis
Ubiquitin mediated proteolysis
Tyrosine metabolism
Taurine and hypotaurine metabolism
Stilbenoid, diarylheptanoid and gingerol biosynthesis
Starch and sucrose metabolism
Plant-pathogen interaction
Phenylpropanoid biosynthesis
Phenylalanine metabolism
Pentose and glucuronate interconversions
Isoquinoline alkaloid biosynthesis
Glycolysis / Gluconeogenesis
Galactose metabolism
Flavonoid biosynthesis
Fatty acid elongation
Diterpenoid biosynthesis
Degradation of aromatic compounds
Biosynthesis of unsaturated fatty acids
Biosynthesis of secondary metabolites
beta-Alanine metabolism
Rich factor
Gene_number
qvalue
NZ19vsZS97- Em
B
Statistics of Pathway Enrichment
Zeatin biosynthesis
Ubiquinone and other terpenoid-quinone biosynthesis
Tryptophan metabolism
Tropane, piperidine and pyridine alkaloid biosynthesis
RNA polymerase
Pyrimidine metabolism
Purine metabolism
Plant hormone signal transduction
Photosynthesis
Phenylpropanoid biosynthesis
Phenylalanine metabolism
Pentose phosphate pathway
Pentose and glucuronate interconversions
Isoquinoline alkaloid biosynthesis
Flavonoid biosynthesis
Fatty acid biosynthesis
Diterpenoid biosynthesis
Cutin, suberine and wax biosynthesis
Biotin metabolism
Biosynthesis of unsaturated fatty acids
Rich factor
Gene_number
qvalue
NZ19vsZS97- Ed
C
Statistics of Pathway Enrichment
Zeatin biosynthesis
Ubiquinone and other terpenoid-quinone biosynthesis
Sulfur metabolism
Starch and sucrose metabolism
Sphingolipid metabolism
Plant-pathogen interaction
Photosynthesis - antenna proteins
Phosphatidylinositol signaling system
Phenylpropanoid biosynthesis
Phenylalanine metabolism
Pantothenate and CoA biosynthesis
Other glycan degradation
Nucleotide excision repair
Inositol phosphate metabolism
Homologous recombination
Galactose metabolism
DNA replication
Diterpenoid biosynthesis
Degradation of aromatic compounds
Brassinosteroid biosynthesis
Rich factor
Gene_number
qvalue
NZ19vsZS97- Al
D
Statistics of Pathway Enrichment
Zeatin biosynthesis
Ubiquinone and other terpenoid-quinone biosynthesis
Tryptophan metabolism
Sulfur metabolism
Stilbenoid, diarylheptanoid and gingerol biosynthesis
Starch and sucrose metabolism
Plant hormone signal transduction
Photosynthesis - antenna proteins
Phenylpropanoid biosynthesis
Phenylalanine metabolism
Other glycan degradation
Glycolysis / Gluconeogenesis
Glutathione metabolism
Galactose metabolism
Flavonoid biosynthesis
Diterpenoid biosynthesis
Degradation of aromatic compounds
Cutin, suberine and wax biosynthesis
Biosynthesis of unsaturated fatty acids
Biosynthesis of secondary metabolites
Rich factor
Gene_number
qvalue
NZ24vsZS97 - Em
E
Statistics of Pathway Enrichment
Zeatin biosynthesis
Ubiquinone and other terpenoid-quinone biosynthesis
Taurine and hypotaurine metabolism
Stilbenoid, diarylheptanoid and gingerol biosynthesis
Starch and sucrose metabolism
Plant hormone signal transduction
Phenylpropanoid biosynthesis
Phenylalanine metabolism
Pantothenate and CoA biosynthesis
Other glycan degradation
Glycosylphosphatidylinositol(GPI)-anchor biosynthesis
Glycosphingolipid biosynthesis - ganglio series
Glycolysis / Gluconeogenesis
Glutathione metabolism
Galactose metabolism
Flavonoid biosynthesis
Fatty acid elongation
Circadian rhythm - plant
Biosynthesis of secondary metabolites
ABC transporters
Rich factor
qvalue
Gene_number
NZ24vsZS97 - Al

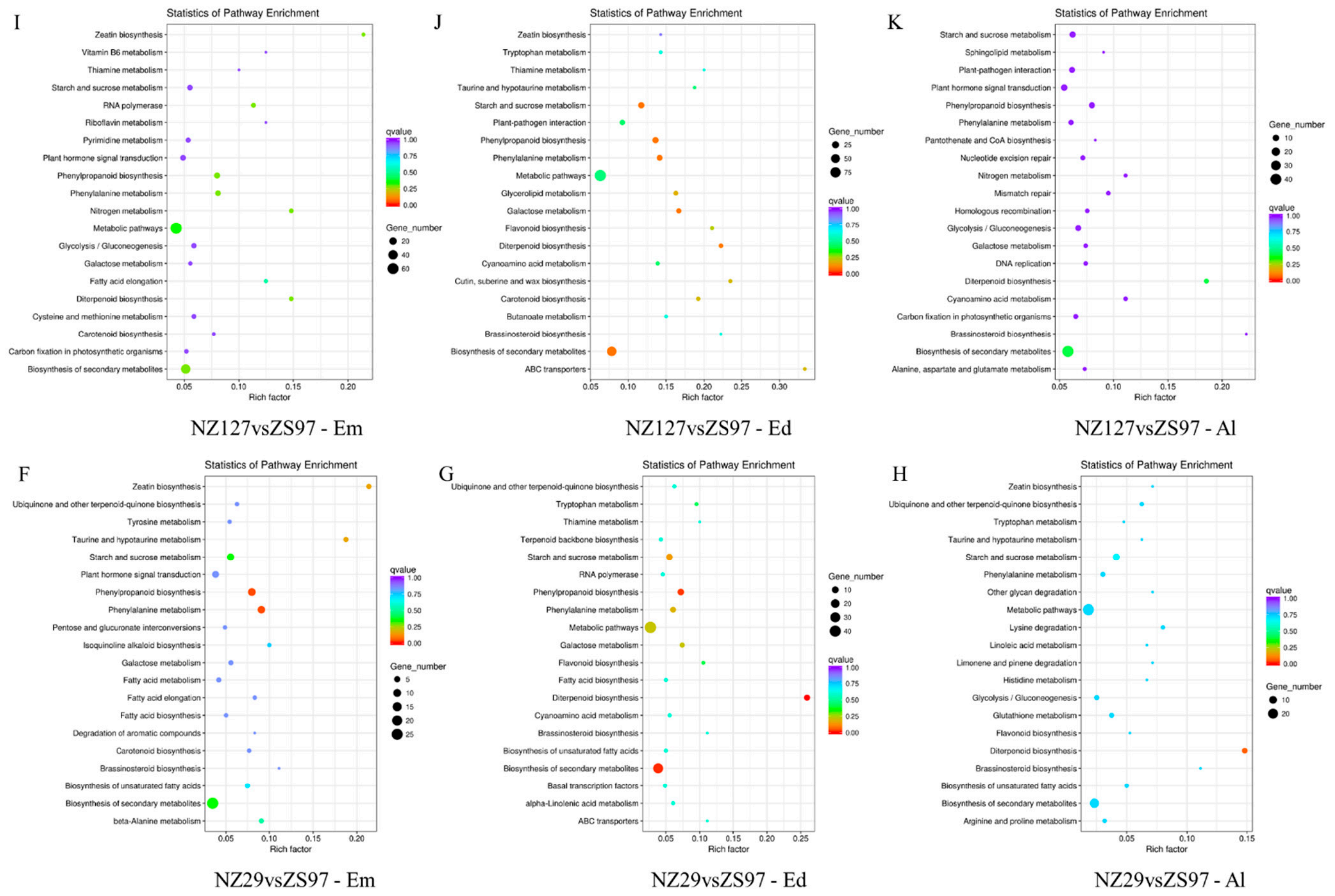

Figure S1. KEGG pathway enrichment analysis of the DEGs in the four non-storable NILs compared with ZS97. Em: embryo; Ed: endosperm; Al: aleurone layer; Rich factor: the ratio of the number of differential genes enriched in the pathway (Sample number) to the number of annotated genes (Background number). The greater the Rich factor, the greater the degree of enrichment.

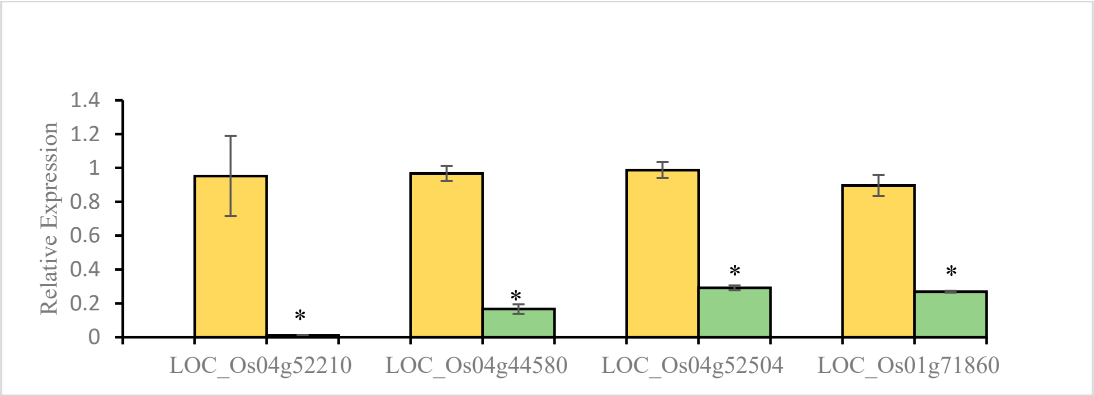

Figure S2. qPCR validation of the selected key genes. Yellow bar represents the relative expression of the corresponding gene in ZS97, and light green bar represents the relative expression of the corresponding gene in the non-storable material NZ127. * indicates significant difference at $p < 0.05$.
